# Identification of Genetic Variation Associated With Heat Tolerance in Cowpea ( *Vigna unguiculata* L. Walp.)

**DOI:** 10.1111/mec.70327

**Published:** 2026-04-03

**Authors:** Roland Akakpo, Elaine J. Lee, Jacob B. Pacheco, Esteban F. Rios, Michael B. Kantar, Ousmane Boukar, Kevin M. Volz, Habib Akinmade, Luis Getino, Kenneth J. Boote, María Muñoz‐Amatriaín, Peter L. Morrell

**Affiliations:** ^1^ Department of Agronomy and Plant Genetics University of Minnesota St. Paul Minnesota USA; ^2^ Agronomy Department University of Florida Gainesville Florida USA; ^3^ Department of Tropical Plant & Soil Sciences University of Hawaii Honolulu Hawaii USA; ^4^ International Institute of Tropical Agriculture Kano Nigeria USA; ^5^ David Volz Design Costa Mesa California USA; ^6^ Plant Breeding Graduate Program University of Florida Gainesville Florida USA; ^7^ Departamento de Biología Molecular Universidad de León León Spain; ^8^ Department of Agricultural and Biological Engineering University of Florida Gainesville Florida USA

**Keywords:** cowpea, environmental genome‐wide association (envGWAS), heat stress, low precipitation, *Vigna*

## Abstract

Heat tolerance is an important trait in cowpea, a crop that is the primary protein source for a large portion of the human population in sub‐Saharan Africa. Cultivated, landrace, semi‐wild, and wild cowpea grow across the region in diverse climatic conditions. This study used environmental association (envGWAS) and allele‐frequency outlier approaches across a panel of 580 gene bank accessions to identify genomic regions associated with adaptation to heat and limited precipitation. Allele frequency outliers are detected independent of potential selective factors driving differentiation; we used a ranking‐based approach to identify the climate variables most associated with variants among outliers. Precipitation‐related variables dominated the signals we identified for envGWAS and allele frequency outliers. We identified variants on all 11 chromosomes that are putatively associated with cowpea adaptation to higher‐temperature environments. The overlap between variants associated with low precipitation and high temperature suggests that these traits may be inextricably linked in cowpea. The Sahel region is the source of many accessions with derived variants associated with high temperatures, suggesting that accessions from this region could contribute heat tolerance alleles for cowpea improvement.

## 1 Introduction

Ongoing environmental change is affecting crop productivity and the stability of food systems (Wheeler and von Braun 2013). Rising global temperatures are making drought and heat the predominant plant stresses (Pereira 2016). As a result, strategies are urgently needed to improve plant adaptation to new climate scenarios (Raza et al. 2019). Crop production is vulnerable to climate change, with rising temperatures among the most significant factors negatively affecting crop yields. Heat stress occurs when temperatures are hot enough for a sufficient period to cause either irreversible damage to plant development and function or an increased rate of reproductive maturation, both of which reduce grain yield (Basra 2023).

Abiotic stresses influence species distribution across diverse environments (Eckhart et al. 2011; Paes de Melo et al. 2022). Plant adaptation to various environmental conditions in response to abiotic stresses often involves multiple survival strategies, including morphological changes, physiological adjustments and molecular responses (Shafi et al. 2022). These adaptive responses encompass a spectrum of reactions ranging from changes in gene expression and physiological adjustments to plant architecture and modifications in primary and secondary metabolism (Mareri et al. 2022). Efforts to identify the genetic basis of plant adaptation to abiotic stress can provide valuable insights into how plants respond to stress, which is crucial for breeding resilient varieties.

An early attempt to find adaptive differences among populations focused on allele frequency differentiation (Lewontin and Krakauer 1973). Efforts to identify the genetic basis of agronomically adaptive traits have focused for many years on bi‐parental crosses and identifying quantitative trait loci (QTL), contributing to the phenotypic variation in segregating populations (Lander and Botstein 1989). Advances in association or linkage disequilibrium (LD) mapping (Lander and Schork 1994) have bridged these divergent approaches (Ross‐Ibarra et al. 2007). Genome‐environment association or environmental genome‐wide association studies (envGWAS) use environmental summaries to identify variants that contribute to adaptation to particular environmental conditions (Bragg et al. 2015; Rellstab et al. 2015; De La Torre et al. 2019). These approaches implicitly assume that measurable environmental variables can be used as proxies for the selective forces shaping genetic differentiation among populations and individuals across geographic space (Tiffin and Ross‐Ibarra 2014). Challenges in applying environmental association include appropriately accounting for population structure (Günther and Coop 2013) and using multiple environmental measurements that result in highly correlated variables (Hoban et al. 2016). There is also the challenge of multiple environmental factors creating interacting selective pressures that shape allele frequencies in ways that do not accord with individual environmental variables (Lotterhos 2023). Nonetheless, in barley ( *Hordeum vulgare* L.), where many large‐effect loci controlling adaptive traits have been identified through QTL and association mapping, envGWAS and allele frequency differentiation approaches have detected most of the known loci (Lei et al. 2019).

Environmental association approaches, combined with complementary analyses such as allele frequency comparisons with F‐statistics and comparisons of allele frequency gradients, have been used in several systems to identify genes likely to explain adaptive differences among populations (Pyhäjärvi et al. 2013; Anderson et al. 2016; Neyhart et al. 2022). In common bean ( *Phaseolus vulgaris* L.), López‐Hernández and Cortés (2019) examined the genetic control of heat tolerance. Although this study used a limited number of wild common bean accessions (78), the authors identified candidate genes related to heat stress response, including heat shock proteins. Moreover, the study found genes involved in multiple biological processes potentially correlated with plant adaptation to high temperatures, including time to flowering, germination and seedling development, cell wall stability and abiotic stress signalling pathways (López‐Hernández and Cortés 2019). In barley, envGWAS, using bioclimatic variables, identified six well‐known loci associated with adaptive variation in flowering time and abiotic stress tolerance (Lei et al. 2019). In maize ( *Zea mays* L.), an envGWAS study examined gene expression responses across diverse environments in Mexico (Gates et al. 2019). This study found loci associated with phenotypic and fitness differences across environments, providing insights into local adaptation. In cowpea ( *Vigna unguiculata* L. Walp.), a recent study performing gene–environment associations on southern African germplasm found three major genomic regions with potential roles in local climate adaptation and candidate genes involved in the regulation of flowering time (Macharia et al. 2025).

In addition to identifying allele frequency differentiation between populations, steep allele frequency gradients have been used to detect adaptive variants (Lasky et al. 2023). Spatial ancestry analysis (SPA) combines locality information and the genotypic state for each sample to identify sharp changes in allele frequency across the landscape (Yang et al. 2012). The direction of allele frequency gradients can differ by variant. This avoids the definition of discrete populations—a long‐standing challenge with *F*‐statistics comparisons (Yang et al. 2012).

Cowpea is an African warm‐season annual legume that provides a valuable source of protein in the diets of millions of people in sub‐Saharan Africa and a nutritious fodder for livestock. Cowpea is also one of the crops listed in the International Plant Genetic Resources Treaty for Food and Agriculture (IPGRTFA) Annex I, which falls under global multilateral benefit‐sharing agreements. The species is well‐adapted to hot and dry environments. Thus, most of the worldwide production is concentrated in drought‐ and heat‐prone zones of sub‐Saharan Africa (Omomowo and Babalola 2021; Abebe and Alemayehu 2022; Affrifah et al. 2022). However, relatively high nighttime temperatures can damage reproductive processes, including flowering, pod formation and seed set, negatively affecting grain yield (Porch and Hall 2013). Previous studies using commercial cowpea varieties have shown linear grain yield reductions of ~5% for every 1°C increase in minimum nighttime temperatures above a threshold of 15°C, up to a 50% grain yield reduction at minimum night temperatures of 26°C (Nielsen and Hall 1985a, 1985b; Hall 2004). In addition, Nielsen and Hall (1985a) showed that increases in minimum nighttime temperature from 16°C to 26°C reduced the pod development period by 7 days, thereby shortening the time to capture resources and decreasing yield. These studies were conducted under long‐day conditions in the subtropics, where nights tend to be colder than those of the major tropical production zones. The photoperiod‐sensitive germplasm, which does not flower under long days, has not been broadly surveyed and could be a reservoir of beneficial alleles for heat stress tolerance.

Extensive genetic resources for cowpea, including landraces and wild relatives, are available from germplasm collections worldwide. These collections harbour functional allelic variation for adaptation to abiotic and biotic stresses. One of the largest cowpea collections is at the International Institute of Tropical Agriculture (IITA), which also holds a Core set of > 2000 accessions that have been genotyped with 51,128 SNPs on the Illumina iSelect Consortium Array (Close et al. 2023).

GWAS approaches using germplasm collections have identified several loci and candidate genes associated with agromorphological and adaptive traits, including pod shattering (Lo et al. 2021), flowering time (Muñoz‐Amatriaín et al. 2021; Paudel et al. 2021), and other phenological traits (Andrade et al. 2023). In addition, QTL mapping in two bi‐parental populations reported QTLs underlying heat tolerance on most cowpea chromosomes (Lucas et al. 2013; Angira et al. 2022).

In this study, we addressed five questions. First, can environmental association approaches identify variants in which allele frequency differentiation suggests adaptation to high‐temperature tolerance? Second, to what extent are variants associated with heat tolerance independent of those identified for adaptation to water deficit? Third, how often do variants identified using mixed model association methods overlap with those found using *F*_ST_ (allele frequency differentiation) and SPA (allele frequency gradient) approaches? Fourth, to what degree are variants identified in *F*_ST_ and SPA analysis associated with extreme temperature and rainfall values based on environmental descriptors of germplasm collection locations? And fifth, given the patterns of linkage disequilibrium in this cowpea collection, to what degree can we identify potential causative loci from the individual variants surveyed?

## 2 Materials and Methods

### 2.1 Germplasm and Passport Data Curation

This study used the IITA Core collection, which includes 2082 worldwide cowpea accessions genotyped at high density (Fiscus et al. 2024). We initially focused on 683 landraces and wild cowpea accessions from Africa with locality information, excluding breeding lines and landraces collected at a ‘market’. Passport data were reviewed in detail, using both manual and automated strategies with the software tool ITALLIC (Onsongo et al. 2022). Inconsistencies were found for 192 accessions, mainly related to mismatches between geographic coordinates and detailed location information. In a coordinated effort with IITA gene bank curators, passport data were updated for 89 accessions, while 103 accessions with insufficient data remained unchanged (Table S1). Among them, a large set of the latter (98 accessions) seemed problematic, as they were categorised as landraces, but all available information suggested they were breeding lines developed by an IITA cowpea breeder in the 1980's. In addition, five other accessions were eliminated, mostly because their location information pointed to a research institute. The curation process resulted in a final count of 580 accessions.

### 2.2 Verifying Geographic Origins

Given the curation required to verify the geographic origin of accessions, we sought additional confirmation of origin by comparing the genotype‐based predictions of initial location with passport coordinates. We used a deep‐neural network tool for spatial ancestry inference (Battey et al. 2020) implemented in the software locator. The inputs for this analysis were iSelect SNP genotyping data and the geographic coordinates for accessions. A randomly selected set of 16 accessions was treated as the query sample, and the remaining accessions were used for training. Uncertainty of sample placement and a 95% confidence interval for sample origin were estimated using 100 bootstrap replicates per query sample.

### 2.3 Extraction of Bioclimatic Data and SNP Curation

A total of 580 cowpea landraces and wild accessions from Africa for which GPS coordinates were accurately determined were included. GPS coordinates were used to extract historical climatic data from WorldClim v2 (http://www.worldclim.com/version2) (Hijmans et al. 2005; Fick and Hijmans 2017). The accessions originated from 30 African countries, with 1 to 173 accessions per country. Passport data, including geographic coordinates, are provided in Table S2.

Genotypic data for 51,128 SNPs were available for these accessions and were curated in Plink 1.9 (Chang et al. 2015) to remove SNPs with > 20% missing data and MAF < 0.02. The physical locations of SNPs were determined relative to the IT97K‐499‐35 genome assembly (Lonardi et al. 2019) using the approach described in (Liang et al. 2024). SNP locations are available in VCF format at https://github.com/MorrellLAB/cowpea_annotation/blob/main/Results/IT97K‐499‐35_v1.0/iSelect_cowpea.vcf.

From ‘WorldClim 2.1’, we extracted bioclimatic data at 30‐s resolution on February 18th, 2023. The getData function from the R package ‘raster’ (Hijmans 2024) was used to extract the data for BIO1 to BIO19 (Table S3). To access the degree of redundancy among the 19 bioclimatic variables, we calculated correlation among them (Figure S1a). In addition, to better clarify the level of independence between heat and drought signals, we quantified the environmental co‐distribution of BIO1 (mean annual temperature) and BIO12 (annual precipitation) and calculated the Pearson correlation of these two variables across sample locations (Figure S1b). We then summarised the 19 bioclimatic information by performing independent component (IC) analysis using the ‘ica’ R package (Durieux et al. 2024), resulting in three additional variables by considering the first three ICs (Table S4). The independent components (ICs) approach is conceptually similar to principal components analysis in summarising data. We also used two other temperature variables corresponding to the maximum and the minimum temperatures associated with the approximate flowering period of the crop in each location, as well as the mean precipitation over the same period. These data were extracted from the WorldClim V2 database using the getData function from the R package ‘raster’ (Hijmans 2024). These three additional variables were obtained by averaging their values over the target flowering period. Altogether, we used 25 environmental variables to identify variants associated with heat tolerance and precipitation.

### 2.4 Structure of Genetic Diversity

Genetic relationships among the 580 cowpea accessions were explored using two strategies. First, genetic assignment implemented in Structure version 2.3.4 (Pritchard et al. 2000) was used to identify the primary population structure in the sample. We tested *K* values from 1 to 8, with 5 replications each. The best *K* value was identified based on the Δ*K* calculation method (Evanno et al. 2005) implemented in the Structure Harvester online program (Earl and vonHoldt 2012). We assigned genotypes to subpopulations based on a membership coefficient (*Q* > 0.75). The ancestry proportions of each sample were represented with a barplot in R. We estimated the spatial extent of each population cluster by projecting the genetic assignment from Structure results on a map of Africa using the tessellation approach of the tess3 R package (Caye et al. 2016). Secondly, the principal component analysis approach available in the R package SNPRelate (Zheng et al. 2012) was used to assess genetic relationships among the accessions.

### 2.5 *F*_ST_ Estimation

The *F*_ST_ estimator (Weir and Cockerham 1984) implemented in the R package ‘HierFstat’ (de Meeûs and Goudet 2007) was used to calculate *F* statistics for each SNP. We used 44,111 SNPs that passed SNP curation and 580 accessions. We considered three partitions of the dataset defined by latitude. The first group included accessions with latitude > 12° N, including samples from Egypt and the Northern Sahel. This latitude corresponds to a transition from equatorial dry winter to hot arid steppe regions and then to hot arid desert, according to the Köppen‐Geiger climate classification (Kottek et al. 2006). The second partition was for accessions with a latitude between 0° and 12° N, including accessions from the Southern Sahara. The third group included accessions with latitude < 0° and comprises accessions from the Southern and Southeastern regions of Africa. Outlier SNPs were identified as the top 0.1% top values of the distribution, that is, 99.9th percentile in each comparison.

### 2.6 SPA Estimation

A spatial ancestry analysis (SPA) (Yang et al. 2012) was used to identify allele frequency gradients across the sampled geographic space. The analysis used genotyping data in PLINK format and geographic coordinates (latitude and longitude) as input. SPA uses a combination of geographic sampling coordinates and allelic states at individual loci to define ‘steep allele frequency gradients’. For biallelic SNPs, this consists of loci where the frequency of two different alleles changes quickly over a relatively small geographic distance. This approach has several advantages. First, it allows us to start with a neutral null hypothesis of isolation by‐distance. Second, allele frequency gradients could occur in any direction across the landscape, and the SPA approach does not require definitions of populations or sample partitions. We identified outlier SNPs as the top 0.1% of the distribution that is, the 99.9th percentile.

### 2.7 Environmental Per‐SNP Genome‐Wide Association Study (envGWAS) and Window‐Based Weighted‐Z Analysis (WZA)

Two GWAS approaches, known for effectively controlling false positives (Cebeci et al. 2023), were used to identify single‐marker associations. In particular, we utilised the multi‐locus GWAS models Fixed and random model Circulating Probability Unification (FarmCPU; Liu et al. 2016) and the Bayesian‐information Linkage Disequilibrium Iteratively Nested Keyway (BLINK; Huang et al. 2019), which are implemented in GAPIT v3 (Wang and Zhang 2021). FarmCPU uses both the fixed‐effects model (FEM) and the random‐effects model (REM) to test markers. BLINK uses the Bayesian information content (BIC) in an FEM and linkage disequilibrium information to test markers. To account for population structure and relatedness, the first five principal components (PCs) computed by GAPIT from the filtered genotype matrix were included as covariates.

We used two datasets: the first included all 580 cowpea accessions, and the second excluded 90 Southeastern accessions, resulting in 490 samples. Environmental GWAS was conducted using 44,111 and 42,399 SNPs, respectively, and the 25 climatic variables described above. Quantile‐Quantile (Q–Q) and Manhattan plots of the *p*‐values were used to evaluate the false positive rate. A false discovery rate (FDR) cutoff of 5% representing the expected proportion of false positives among the rejected hypotheses was used to identify significant associations for each of the 25 climatic variables. Using the R package *q*‐value version 2.30.0 (Storey et al. 2022), *q*‐value was computed from SNP *p*‐values, and SNPs with *q*‐value < 0.05 were considered significantly associated with the trait (BIO1–BIO19, TMAX, TMIN, PREC, IC1, IC2 and IC3), corresponding to the expected FDR of 0.05.

We performed a window‐based genotype–environment association analysis to identify genomic regions associated with environmental variables in cowpea (Booker et al. 2024). WZA was applied using 50 kb non‐overlapping windows across the genome, under the assumption that selection causes LD and clustered signals. For each window, a *Z*‐score and corresponding *p*‐value were computed from the aggregated association statistics of the individual SNPs within the window. Outlier windows were defined as the top 1% of windows with the smallest *Z*‐score *p*‐values (i.e., the 99th percentile). To identify high‐confidence SNPs within candidate windows, we intersected the candidate window with per‐SNP association results.

### 2.8 Ranking of Environmental Variables

We used three temperature (BIO1, BIO5 and BIO8) and three precipitation‐related variables (BIO12, BIO13 and BIO16) to rank all georeferenced accessions relative to their ordinal value for each bioclimatic variable. We used this approach to identify putative environmental factors associated with the presence of the alternate allele in our *F*_ST_ and SPA analyses. For instance, BIO1 was reported as the average annual temperature expressed as a floating‐point number at each location. This value can be sorted so that each accession is assigned a discrete ordinal value based on its rank in our sample list. Thus, based on the rank order of values per SNP, we could compare which bioclimatic variables were most associated with alternate alleles for allele frequency outliers. We finally evaluated whether rank distributions differed significantly across variables using a Friedman nonparametric chi‐square test (Friedman 1937). This test identifies cases in which one or more environmental variables exhibit consistently higher rank values than expected by chance, indicating a stronger association between a given bioclimatic variable and an allele‐frequency outlier.

### 2.9 Linkage Disequilibrium, Variant Annotation and Enrichment Analysis of Hits

We used Plink 1.9 (Chang et al. 2015) to estimate the level of LD in our dataset. Pairwise *r* ^2^ was calculated between SNPs on the same chromosome using the full genotyping dataset. Values for *r* ^2^ were plotted as a function of physical distance. LD decay was visualised with a scatterplot and smoothed curve, and the distance at which *r* ^2^ decayed to half its initial value was used to summarise the decay rate.

Annotation for iSelect variants based on the *V. unguiculata* genome (IT97K‐499‐35_v1.0) (Lonardi et al. 2019) using Variant Effect Predictor (VeP) (McLaren et al. 2016) was reported by (Liang et al. 2024), and it is available at: https://github.com/MorrellLAB/cowpea_annotation/tree/main/Results/IT97K‐499‐35_v1.0/VeP. SNPMeta (Kono et al. 2014), which performs BLAST (Camacho et al. 2009) parsing to extract annotations from the current versions of the National Center for Biotechnology Information (NCBI) GenBank nucleotide database (Sayers et al. 2024), was used to identify gene names and the identity of annotated copies of genes containing each of our outlier SNP in related species.

Based on envGWAS, *F*_ST_ and SPA outlier SNPs, we calculated the odds ratio for enrichment in genic regions, coding sequences (CDS), and nonsynonymous variants (Findley et al. 2021). The odds ratio was used to determine whether these variant categories were significantly enriched in the outlier dataset relative to the genomic background of the full genotyping data. We then applied a Fisher's exact test to evaluate the significance of each observed enrichment.

### 2.10 Inference of the Ancestral State of Variants

In most cases, new adaptive functions can be attributed to derived mutations. To infer the derived versus ancestral state at each SNP, we used a maximum likelihood method (Keightley et al. 2016), updated to use multiple outgroup species and nucleotide substitution models (Keightley and Jackson 2018) and implemented in the program est‐sfs (https://sourceforge.net/projects/est‐usfs/). We used genome assemblies of three *Vigna* species as outgroup sequences. These included *V. vexillata* (Zombi pea), *V. marina* (beach pea or notched cowpea) and *V. triolobata* (jungle mat bean) (Naito et al. 2022). We used phylogenetic relationships as estimated from nuclear ribosomal and chloroplast genes (Takahashi et al. 2016). We aligned the assemblies to the *V. unguicalata* genome (IT97K‐499‐35_v1.0) (Lonardi et al. 2019) using minimap2 v2.28 (Li 2018) (https://github.com/MorrellLAB/cowpea_annotation). The resulting sequence alignment map (SAM) files were sorted, indexed and converted to a binary alignment map (BAM) file using samtools v1.20 (Li et al. 2009). The alignments for each outgroup accession were converted to FASTA files using the analysis of the next‐generation sequencing data (ANGSD) v0.940 function ‘doFasta’ (Korneliussen et al. 2014). The nucleotide state at SNP positions was extracted using the bedtools v2.31.0 function ‘getFasta’ (Quinlan and Hall 2010). Input files for ancestral state inference included a VCF of the focal species and BED files of the inferred nucleotide state. Input and output for the analysis were processed using Python scripts available at https://github.com/MorrellLAB/File_Conversions. The program est‐sfs v2.04 was run using the six‐rate nucleotide substitution model reported by Keightley and Jackson (2018), designated R6. The program uses allele‐frequency information from the focal species and nucleotide‐state information from each outgroup species to estimate the probability that the major allele at each variant is the ancestral state. The program reports the probability that the major allele is ancestral. For probability values > 50%, we reported the raw probability and the major allele. For probabilities *p* < 50%, we reported 1‐*p* and the minor allele. We reported *p* > 80% as the ancestral allele in an annotated VCF file. All inferred ancestral states and associated probabilities are available (along with the VCF) in a Data Repository for the University of Minnesota (DRUM) (https://doi.org/10.13020/XXPS‐Q694).

## 3 Results

### 3.1 Accession Locality Confirmation

**FIGURE 1:**
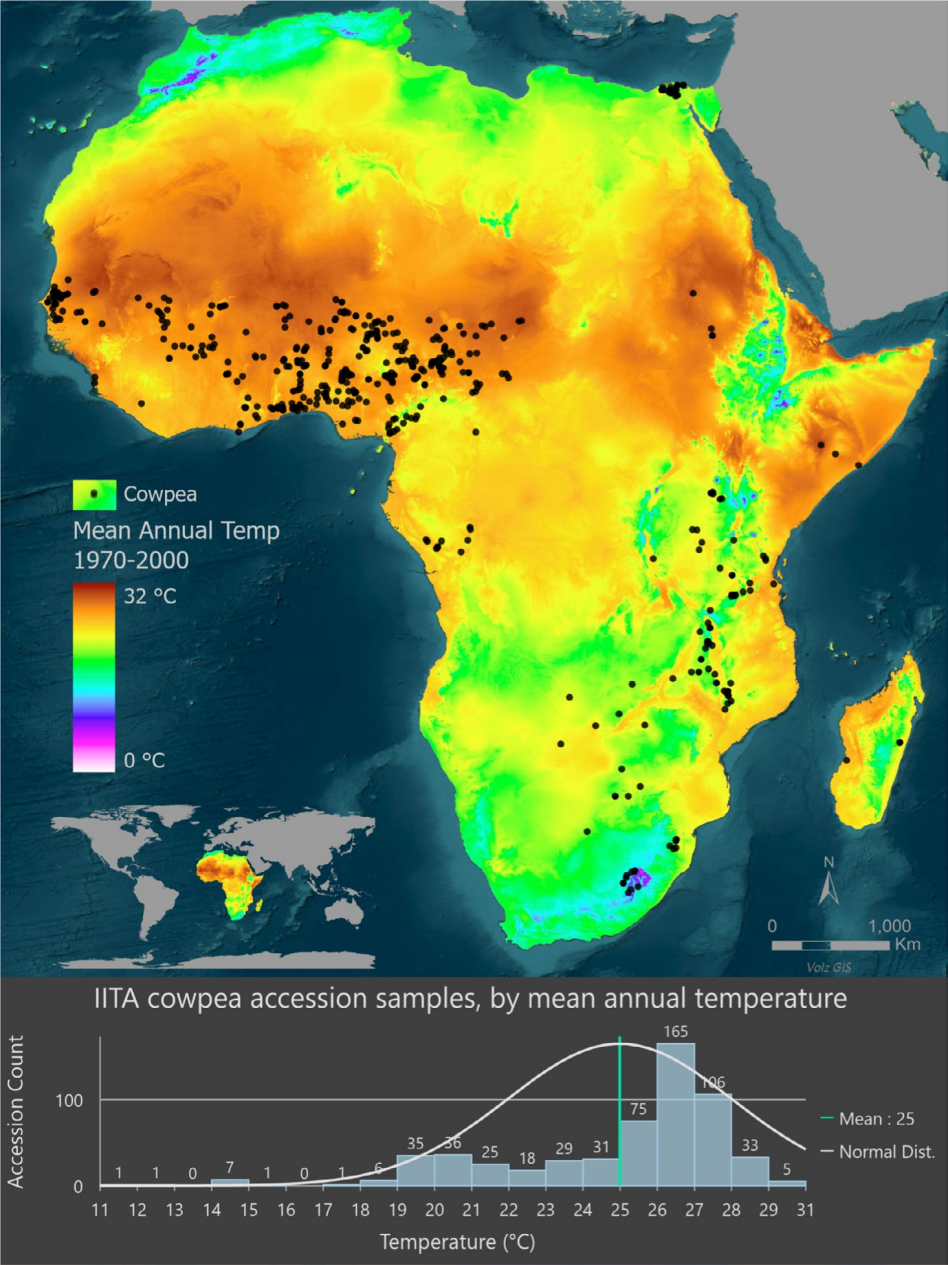
The map depicts the average mean annual temperature from 1970 to 2000. The map shows the collection site of 580 cowpea accessions as black dots. Darker dots indicate multiple accessions in the same area. The mean annual temperature was extracted from WorldClim v2. The histogram below shows the frequency distribution of accessions according to the mean annual temperature at their collection sites.

The germplasm used in this study is a subset of the IITA cowpea core collection. A set of 683 accessions from Africa was initially selected, primarily categorised as landraces (632 accessions). This sample set excluded accessions where the ‘collection site’ was a market. The accuracy of geographical information within the passport data is crucial for the success of the envGWAS approach. A detailed exploration of the passport data of the 683 accessions, including the use of the geographic sampling confirmation program ITALLIC (Onsongo et al. 2022), revealed inconsistencies in geographic origin information for 190 accessions. A detailed curation of the passport data was performed (see Methods, Figure S2 and Table S1 for details), which resulted in a final set of 580 accessions for further analysis. The 580 accessions had been previously genotyped with 51,128 SNPs on the Illumina iSelect Cowpea Consortium Array (Muñoz‐Amatriaín et al. 2017), and the genotypic data are available from Close et al. (2023). These accessions were mapped onto the African continent to compare with the mean annual temperature from 1970 to 2000 obtained from the WorldClim2 database (Fick and Hijmans 2017) (Figure 1). The accessions occur across a relatively large temperature and precipitation gradient, consistent with differentiation into very distinct climatic zones (Figure 1; Figure S3). Based on the current Köppen‐Geiger classification (Kottek et al. 2006), most accessions in Western Africa occur in the tropical savanna climatic zone (Figure S3). Most accessions from the hottest climates in Africa occur in the tropical savanna, with a small number occurring in the hot desert climate on the southern edge of the Sahara.

### 3.2 Population Structure

**FIGURE 2:**
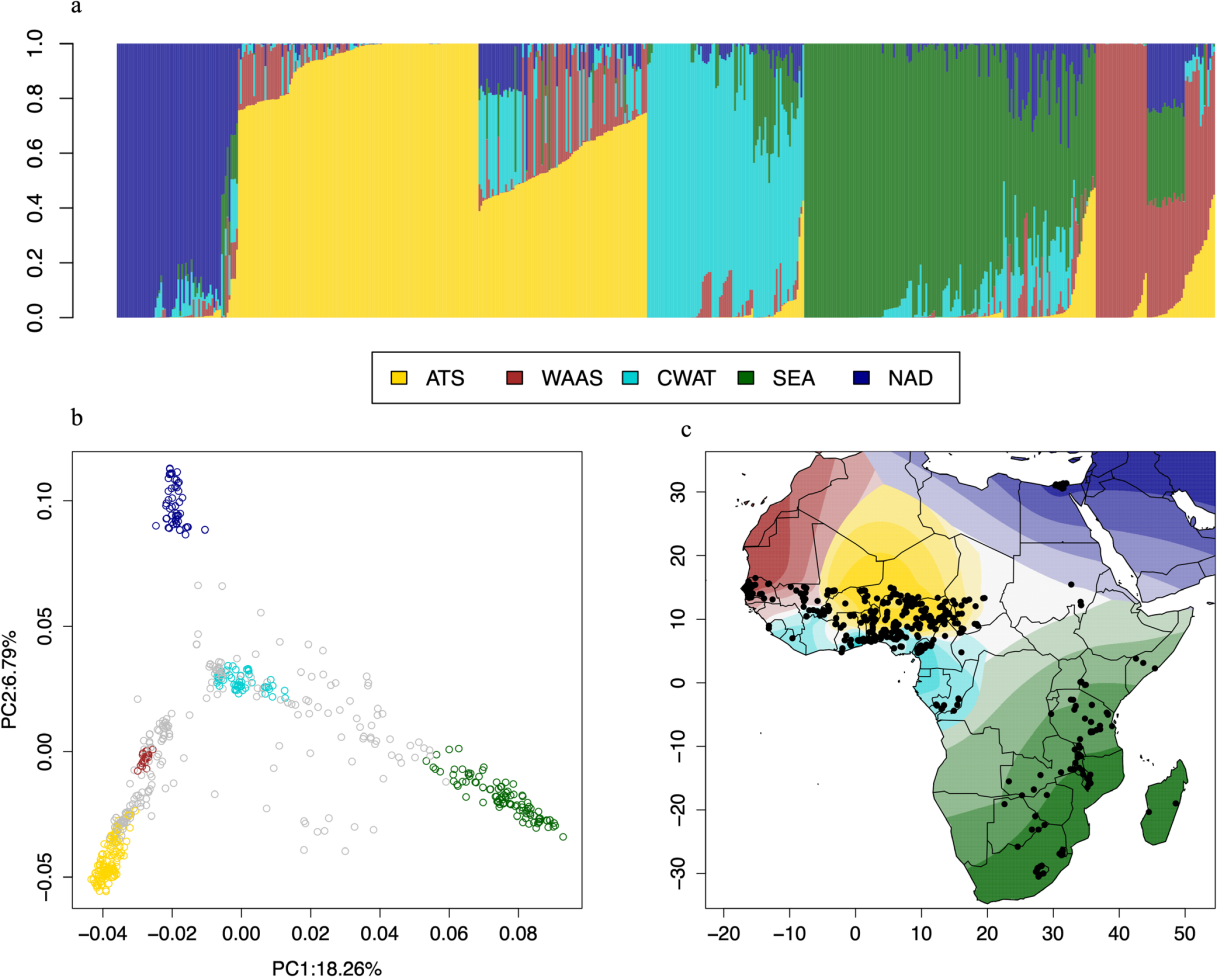
(a) Genetic assignment results for *K* = 5. ATS = African Tropical Savannah (Yellow), CWAT = Coastal West African Tropical (Light blue), NAD = North African Desert (Dark blue), SEA = Southeastern Africa (Green), WAAS = West African Arid Steppe (Red). (b) Principal Component Analysis (PCA) showing genetic clustering; (c) Spatial interpolation of the geographic distribution of genetic clusters.

Population structure in the dataset was assessed using genetic assignment in the software Structure (Pritchard et al. 2000) and by a principal component analysis (PCA). Using Structure outputs for a range of population numbers (*K*) from 1 to 8, Δ*K* values (Evanno et al. 2005) were calculated. Δ*K* reached a maximum at *K* = 2, which primarily distinguished Southeastern African accessions from the balance of the sample, with a secondary peak at *K* = 5 (Figure S4). *K* = 5 was used for further analysis as it provided a more detailed description of the genetic structure in this diverse dataset (Figure 2a). We assigned samples to populations based on ancestry proportions (*Q*) > 0.75. The first genetic cluster comprised 127 accessions primarily from the African Tropical Savannah (ATS), the hottest growing region in our dataset. A second cluster included 27 accessions derived primarily from the West African Arid Steppe (WAAS), including Senegal and Gambia. A third cluster included 56 accessions from the Coastal West African Tropical area (CWAT). A fourth cluster included 105 accessions primarily from Southeastern Africa (SEA), while the fifth cluster comprised 55 accessions mostly from locations in the North African Desert (NAS), especially Egypt.

A principal component analysis (PCA) identified similar populations (Figure 2b, Figure S5). Together, PC1 and PC2 (Figure 2b) explain 25% of the total genetic variation. The first principal component separated the samples from Southern Africa (SEA) from the samples belonging to other subpopulations. The second principal component separated samples belonging to Egypt and Northern Africa (NAS) from accessions belonging to populations from western Africa (ATS) and those from the coastal region of Africa (WAAS), and to a lesser degree, accessions from western Africa (CWAT) (primarily from tropical savanna regions) are intermediate between these two extremes.

Genetic assignment based on Structure results was projected on a map of Africa using the tessellation approach implemented in Tess3 (Caye et al. 2016) (Figure 2c).

Based on the identified population structure, indicating a strong genetic differentiation of the Southeastern African germplasm from the rest, which also correlates with temperature (i.e., samples from Southeastern Africa are adapted to lower growing temperatures, Figure 1), we eliminated 90 Southeastern Africa samples for most of our further analysis and focused on the remaining 490 samples. These primarily included accessions from Western Africa, with a few samples from adjacent regions and Egypt.

### 3.3 Identification of Loci Putatively Associated With Environmental Adaptation

We used frequency‐based measures and a mixed model association approach (envGWAS) to identify variants potentially associated with heat tolerance in cowpea. A total of 297 individual SNPs were identified following the workflow described in Figure S6.

#### 3.3.1 Allele Frequency ( *F*_ST_ ) Outliers

We examined allele frequency differentiation for three geographic partitions of cowpea accessions. We first used the sample set of 490 samples, excluding Southeastern African accessions, and compared samples occurring at > 12° N latitude—primarily in the Köppen‐Geiger climate classes Bwh (hot desert) or Bsh (semi‐arid climates) (Kottek et al. 2006) that are exposed to warmer and drier growing conditions than accessions from lower latitudes in West Africa (Figure S3). Accessions from equatorial regions are primarily from climate zone Aw (tropical wet savanna climate), with a small number of accessions from Am (tropical monsoon climate) (0° N < latitude < 12° N) (Figure S3). This resulted in 289 of 490 accessions in this sample that occurred north of the > 12° N latitude comparison. Genome‐wide average *F*_ST_ = 0.039 (± 0.062), with a median of 0.011. Genetic differentiation, as measured by *F*_ST_, was greater towards the ends and lower towards the middle of each chromosome, but we did not identify any genomic regions with exceptionally high *F*_ST_ values. Using an empirical cutoff of the 99.9th percentile of *F*_ST_ values, we identified 44 SNPs with *F*_ST_ ranging from 0.366 to 0.459 (Table 1; Table S5). The average minor allele frequency (MAF) of our *F*_ST_ outliers was 0.279 (± 0.058), consistent with the constraint on *F*_ST_ towards higher *F*_ST_ values at higher MAF.

**TABLE 1:** The 44 SNPs identified as outliers in *F*_ST_ analysis.

SNP	Chromosome	Position	REF	ALT	MAF	*F*_ST_
2_41147	Vu01	35265694	T	C	0.2691	0.367421642
2_23980	Vu02	18984962	A	C	0.2163	0.373396979
2_25308	Vu02	20227677	G	T	0.3673	0.371434409
2_07556	Vu02	20244870	T	C	0.2229	0.365564798
2_40702	Vu02	2161789	T	C	0.2327	0.447939419
2_25127	Vu03	55523972	A	G	0.3622	0.36707004
2_22916	Vu03	38231022	T	C	0.2531	0.368525334
2_00372	Vu03	38214324	T	G	0.3163	0.375015544
2_30891	Vu04	14635703	A	G	0.2163	0.366013666
2_40224	Vu04	24273744	T	C	0.3673	0.404123228
2_47878	Vu04	12434298	T	C	0.2691	0.383600433
2_40290	Vu04	12460174	C	T	0.2327	0.389740721
2_19601	Vu05	3802677	G	A	0.2531	0.407273132
2_18508	Vu05	3708611	C	T	0.2229	0.368754761
2_53903	Vu05	3739351	A	C	0.3163	0.404886057
2_25041	Vu05	3835894	G	T	0.3622	0.430975934
2_27980	Vu05	5936454	G	T	0.2691	0.365169439
2_08871	Vu05	5942506	T	C	0.2327	0.392285478
2_28186	Vu07	26109238	C	T	0.2163	0.388022533
2_21861	Vu07	26137988	A	G	0.3673	0.388022533
2_48714	Vu07	26149694	C	T	0.2229	0.38890675
2_49476	Vu07	26156897	T	C	0.3163	0.388022533
2_25770	Vu07	26168611	C	T	0.2531	0.440645803
2_03060	Vu07	26173924	A	G	0.3622	0.442324938
2_00885	Vu08	34707621	A	C	0.2327	0.404014544
2_07072	Vu08	30038329	C	T	0.3622	0.369921954
2_00857	Vu08	7392202	T	G	0.2691	0.366142054
2_09973	Vu08	7405223	C	A	0.2327	0.366142054
2_29223	Vu08	7406627	C	T	0.2163	0.372196772
2_51987	Vu08	7408089	C	T	0.3673	0.372196772
2_53354	Vu08	7412329	T	C	0.2229	0.372872684
2_51407	Vu08	7419005	C	T	0.2531	0.366446259
2_52823	Vu08	7417594	A	G	0.3163	0.378947462
2_06639	Vu08	30099911	A	G	0.2691	0.365935158
2_51380	Vu09	38945148	C	T	0.3673	0.451429091
2_05643	Vu09	41247766	A	G	0.3163	0.384307282
2_25481	Vu09	41181696	C	T	0.2229	0.384554079
2_14464	Vu09	41270428	C	A	0.2531	0.386258619
2_14668	Vu09	31846740	T	C	0.2163	0.37395871
2_19641	Vu11	39324907	C	A	0.3673	0.428637588
2_25564	Vu11	35441960	G	A	0.3622	0.458509445
2_17698	Vu11	39316305	T	C	0.2163	0.398862745
2_02052	Vu11	35816995	G	T	0.2691	0.39222921
2_02051	Vu11	35817750	C	A	0.2327	0.39222921

**FIGURE 3:**
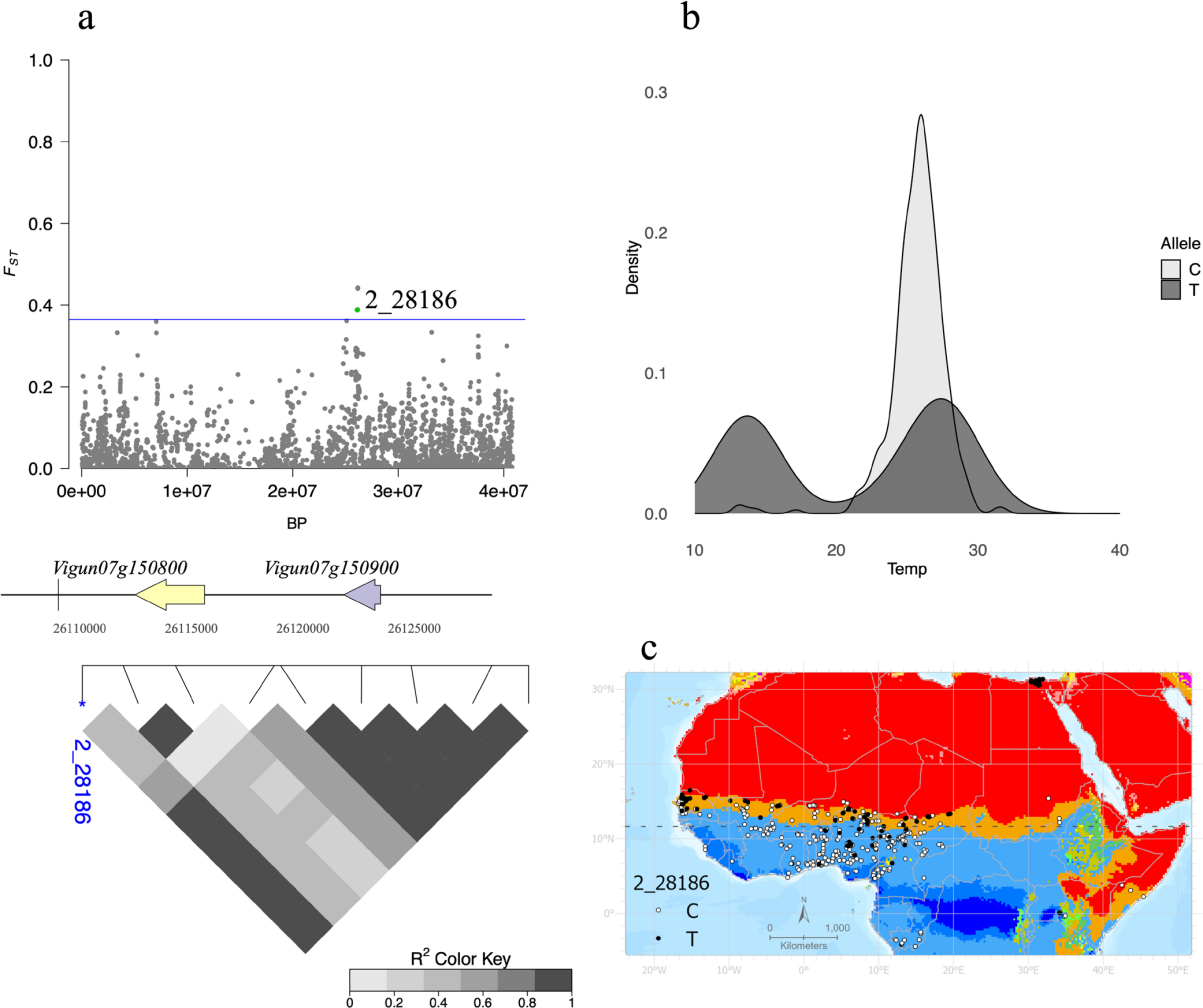
(a) *F*_ST_ values for Vu07 are depicted in a Manhattan plot. The two genes in closest proximity to SNP 2_28186 are depicted, along with pairwise LD for nine neighbouring SNPs. The closest annotated ortholog in *Glycine max* corresponds to cyclin‐L1‐1‐like isoform X2. (b) The distribution of 2_28186 alleles across a range of temperatures. (c) The geographic distribution of alleles (C and T) at SNP 2_28186 in West African cowpea accessions.

The highest *F*_ST_ values included 10 SNPs on chromosome Vu08; seven occurred within a 27‐kb genomic region that includes two genes (*Vigun08g058300* and *Vigun08g058400*). The second‐largest block of high *F*_ST_ variants included six SNPs in a 65‐kb region on chromosome Vu07. The first of these six SNPs, from the 5′ end of the region was SNP 2_28186 (Figure 3a,b), where the alternate and derived allele was found most abundantly in samples from the western Sahel region (Figure 3c). Near the beginning of the region (~26,113,000 bp), are two genes encoding DNAJ heat shock proteins, *Vigun07g150800* and *Vigun07g150900* (Figure 3a). DNAJ has been described as a heat shock protein that plays a key role in the growth and heat stress response of chilli peppers ( *Capsicum annuum* L.) (Fan et al. 2017).

For the six variants (2_28186, 2_21861, 2_48714, 2_49476, 2_25770, 2_03060) on Vu07, the average MAF is = 0.29, with alternate alleles that occur in accessions across a broad geographic range, including the hottest localities. Using a series of nine *F*_ST_ outliers, we identified relatively high levels of linkage disequilibrium in the ~65 kb region, with an average *r* ^2^ of 0.62 (Figure 3a).

*F*_ST_ comparisons between samples from the African equatorial region (0° N < latitude < 12° N) and those from Southern Africa (latitude < 0° N) resulted in much higher values (mean *F*_ST_ = 0.231 ± 0.180, median = 0.201). A comparison of populations from higher latitudes (> 12° N) to Southern African accessions resulted in *F*_ST_ = 0.197 (±0.149) with a median of 0.181 (Figure 3a,b). The demographic history of cultivated cowpea has not been extensively explored. Still, elevated *F*_ST_ values when compared with populations from Southern Africa could suggest very different demographic histories for cultigens across various portions of the species' range in Africa.

#### 3.3.2 Allele Frequency Gradients (SPA)

**FIGURE 4:**
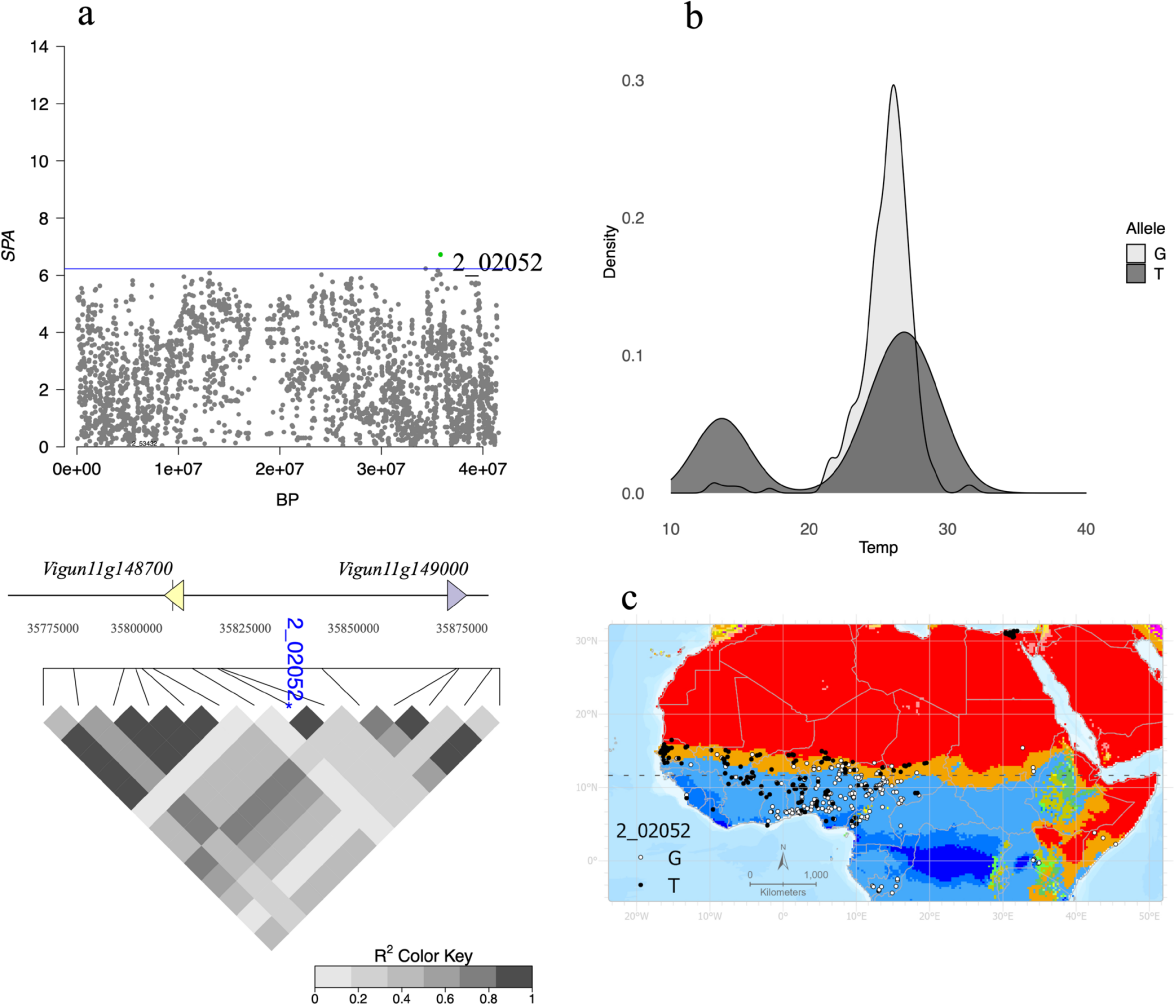
(a) SPA values for Vu11 are depicted in a Manhattan plot. The 2 genes in closest proximity to SNP 2_02052 are depicted along with pairwise LD for 14 neighbouring SNPs. The closest annotated ortholog in *Glycine max* corresponds to ethylene‐responsive transcription factor RAP2‐7 isoform X1. (b) The distribution of alleles at 2_02052 is the highest value of SPA outlier. (c) Geographic distribution of alleles (G and T) at SNP 2_02052 in Western African cowpea accessions.

Using the same empirical cutoff at the 99.9th percentile for 42,339 SNPs, the SPA analysis identified 42 outliers with SPA values > 6.227 (Table 2). SPA values ranged from 0.002 to 6.725, with a median of 1.814 and a mean of 2.123 (±1.459). The largest SPA values were observed for two SNPs (2_02051 and 2_02052) on Vu11, which were also identified as outliers in the *F*_ST_ analysis (Figure 4a,b). The two SNPs followed a nearly identical geographic pattern, with the alternate (and ancestral) alleles most abundant in West Africa and becoming less common in cooler, wetter climates to the southeast (Figure 4c). Both SNPs occurred in introns of the gene *Vigun11g148700* (Figure 4a), annotated as an ethylene‐responsive transcription factor, which belongs to a gene family previously associated with heat stress response in *Arabidopsis thaliana* (Huang et al. 2021). High LD values were also observed in that same region on Vu11. A series of 14 SPA outliers on chromosome Vu11 spanned a region of ~106 kb with a mean *r* ^2^ value of 0.44 (Figure 4a). But the largest group of SPA outliers occurred in 11 of the 12 SNPs, on a Vu08 region spanning 352 kb. This region includes 50 genes and does not overlap with variants found by envGWAS or *F*_ST_ outlier analyses.

**TABLE 2:** The 42 SNPs identified as outliers in SPA analysis.

SNP	Chromosome	Position	REF	ALT	MAF	SPA
2_40911	Vu02	9772009	C	T	0.119388	6.33218595
2_41585	Vu02	13201161	C	T	0.131633	6.34684379
2_15286	Vu02	32217978	C	T	0.130612	6.38583729
2_31898	Vu02	33609908	G	T	0.119388	6.26171032
2_22825	Vu02	33732878	T	G	0.138776	6.26811305
2_42020	Vu04	5583939	C	T	0.118896	6.53341899
2_40290	Vu04	12460174	C	T	0.718367	6.30143805
2_40224	Vu04	24273744	T	C	0.158436	6.30983993
2_11664	Vu05	366583	T	C	0.152041	6.48889183
2_30578	Vu05	371882	G	A	0.156122	6.4797115
2_06865	Vu05	618953	C	T	0.126531	6.46012734
2_49015	Vu05	761491	G	A	0.127551	6.44281203
2_54794	Vu05	1373737	C	T	0.189331	6.28440948
2_16071	Vu05	3728035	T	C	0.130612	6.30292467
2_05074	Vu05	3738124	A	G	0.121429	6.361751
2_07002	Vu05	12163327	G	T	0.403974	6.31524117
2_25170	Vu05	46120114	G	A	0.115942	6.43666647
2_18368	Vu05	47493777	A	G	0.134969	6.43587753
2_30853	Vu07	3369363	G	T	0.12449	6.62377586
2_02754	Vu07	5310677	A	G	0.126294	6.25800453
2_46140	Vu07	5721012	T	C	0.137149	6.42867755
2_25770	Vu07	26168611	C	T	0.222904	6.3098184
2_03060	Vu07	26173924	A	G	0.216327	6.35519846
2_16556	Vu08	255408	A	G	0.129592	6.34855549
1_0684	Vu08	263699	C	A	0.126531	6.49674083
2_29455	Vu08	273444	A	G	0.152083	6.2905076
2_23087	Vu08	292459	T	G	0.129592	6.25917809
2_01893	Vu08	394592	G	A	0.135714	6.36260038
2_15263	Vu08	410767	C	A	0.139706	6.33953461
2_15262	Vu08	411790	C	T	0.135714	6.34272106
2_33384	Vu08	416063	A	T	0.156122	6.23319836
2_31016	Vu08	416227	A	C	0.156122	6.23319836
2_04503	Vu08	420378	G	T	0.161224	6.22784024
2_09210	Vu08	607696	T	C	0.127551	6.27960781
2_32541	Vu08	27486972	C	G	0.118367	6.53221898
2_14338	Vu09	29038851	C	T	0.130612	6.33701787
2_15819	Vu09	32114933	T	G	0.877551	6.37556006
2_26718	Vu09	34064769	A	G	0.881633	6.33974334
2_15395	Vu10	37534007	C	T	0.12449	6.36096369
2_50899	Vu11	34351838	C	T	0.169388	6.23215601
2_02052	Vu11	35816995	G	T	0.383673	6.72527267
2_02051	Vu11	35817750	C	A	0.383673	6.72527267

For SPA outliers, the potential nature of selective factors in steep allele frequency gradients is less evident because, unlike *F*_ST_ comparisons, the observed allele frequency differentiation occurred in many different geographic partitions of the sample. Comparing SPA outliers to temperature and precipitation environmental variables at the localities that carry the minor allele identified, for most of the SPA outliers, annual precipitation (BIO12) had the lowest rank values, suggesting that precipitation drives the allele frequency gradients (Figures S7 and S8). The only exceptions were three SNPs (2_02051, 2_02052 and 2_07002) in which the maximum temperature of the warmest month (BIO5) ranked lowest (Table S4).

#### 3.3.3 Environmental Genome‐Wide Association Study

We used the multi‐locus mixed models FarmCPU and BLINK implemented in the R package GAPIT3. The first five PCs explaining ~36% of the total genetic variation were included as covariates in each model, to investigate 25 climatic variables including 22 bioclimatic variables and their three summary Independent Components (IC1, IC2 and IC3), for the environmental association. These independent components summarised the major climatic gradients, with IC1 capturing precipitation during the wettest period, IC2 representing precipitation during the driest period, and IC3 reflecting overall temperature patterns that were subsequently used to define heat‐associated SNPs (Table S4). We used the false discovery rate (FDR) control at 5% to detect significant associations based on *q*‐value calculation. As bioclimatic variables are highly correlated (Figure S1a), we focused only on significant SNPs (*q*‐value < 0.05), associated with at least two temperature or precipitation variables. Using 490 samples, excluding those from southeastern Africa, we identified 89 SNPs associated with temperature and precipitation variables. When using all 580 accessions in the dataset, we detected 149 SNPs associated either with temperature (or TEMP) variables (BIO1 to BIO11 + IC3, mean MAF = 0.131 ± 0.115) or precipitation (PREC) variables (BIO12 to BIO19 + IC1 + IC2, mean MAF = 0.134 ± 0.132) (Table S5). We summarised the variables identified into five groups. The TEMP group includes 87 SNPs associated with BIO1—BIO11 or IC3. The PREC group includes 149 SNPs associated with BIO12—BIO19 or IC1 and IC2. The HIGH (temperature) group has 11 SNPs associated with BIO5, BIO9 or BIO10. The DRY group contains 24 SNPs associated with either BIO14, BIO17, or BIO18. Finally, the HEAT group includes 11 SNPs associated with TMIN or TMAX. Altogether, we identified 217 unique SNPs (i.e., 19 SNPs were detected in both datasets, and some of them were associated with more than one variable) (Table S5). Manhattan and quantile–quantile plots were assessed visually for representative envGWAS runs, to support interpretation. All these plots are available in the GitHub repository (https://github.com/MorrellLAB/cowpea_environmental/tree/main/01_envGWAS/Results).

For TEMP hits, some genomic regions stood out, with individual SNPs distributed across chromosomes Vu04, Vu05, Vu07, Vu10 and Vu11 (MAF < 5%). These hits were also associated with the PREC variables and could be useful in cowpea breeding for high‐temperature tolerance.

A window‐based weighted *Z* analysis using a 50 kb window across the genome identified 617 outlier windows, exceeding the 99th percentile of the genome‐wide WZA distribution, thus potentially associated with cowpea environmental variables. Among these 617 outlier windows, 34 overlapped 39 of the 297 outliers SNP identified by per‐SNP analyses, indicating a similarity in the genomic signal despite the difference in approaches. All WZA output files including windows statistics and ranking are available in the GitHub repository: (https://github.com/MorrellLAB/cowpea_environmental/tree/main/01_envGWAS/WZA).

### 3.4 Environmental Variables Ranking

Allele‐frequency differentiation identified loci with variant frequencies between regions but provided limited information about the potential nature of differences between localities that could induce selective changes in allele frequency. We used three temperature and three precipitation‐related bioclimatic factors that can be ranked from most to least extreme value to determine if these factors could explain allele frequency differences at outlier loci, based on *F*_ST_ and SPA results. Temperature values for accessions were sorted such that the highest temperature or the lowest precipitation values had the highest ranks. For samples with elevated *F*_ST_ values, the maximum temperature of the warmest month (BIO5) ranked lowest among all outliers. The second lowest‐ranked variable was the mean temperature of the wettest quarter (BIO8). All results are in the GitHub repository.

### 3.5 Overlap of Variants Detected

**FIGURE 5:**
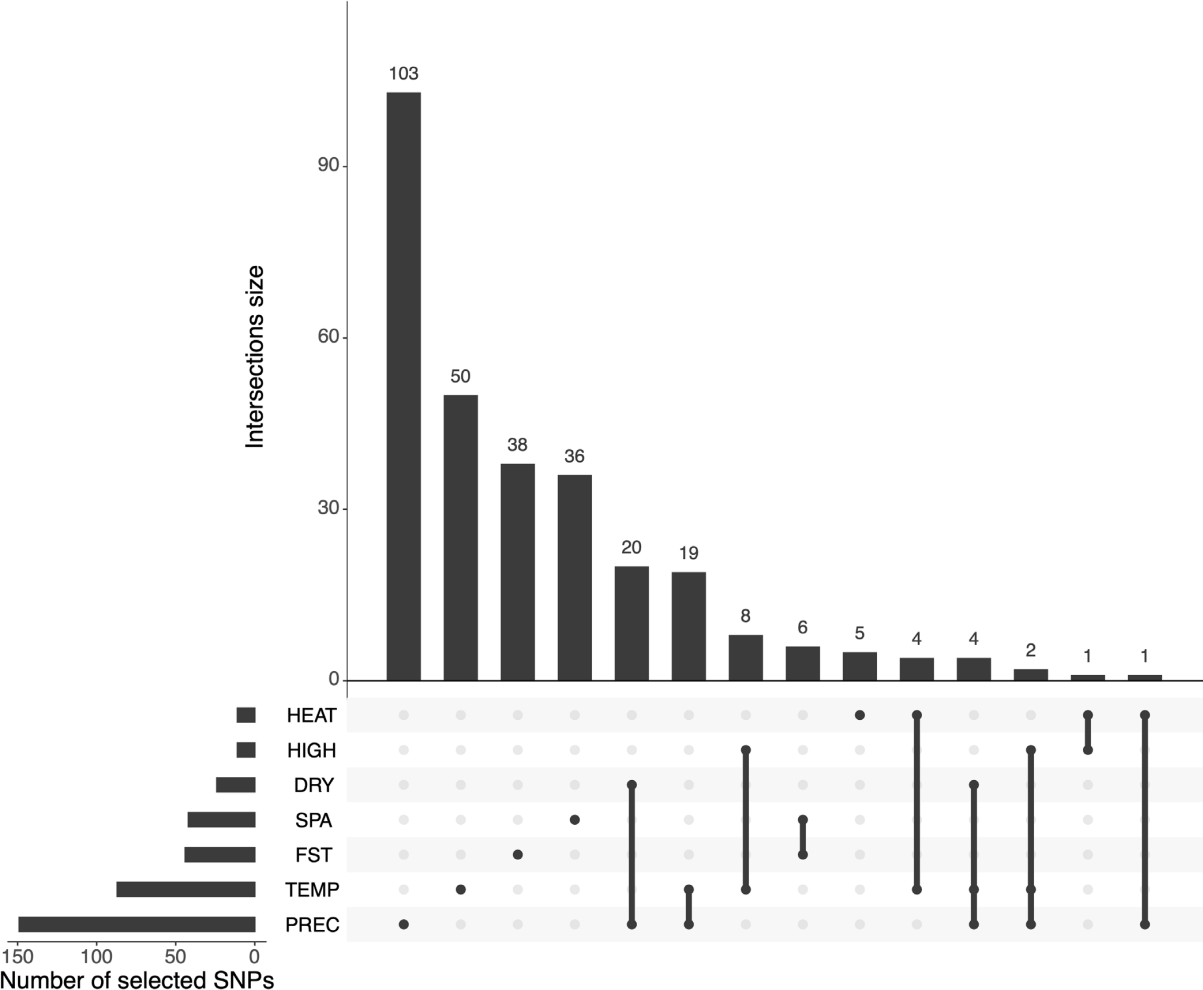
Upset plot showing the overlap of variants between classes of climatic variables and allele frequency approaches. Many SNPs are shared across multiple climatic categories. Horizontal bars show the number of SNPs per class of climatic variables; vertical bars present the size of the overlap of variants. The black dot indicates the climatic variables classes contributing to each overlap shown in the vertical bar.

We found extensive overlap for variants identified in envGWAS associated with TEMP and PREC (Figure 5). However, none of the variants identified in the envGWAS overlapped with those detected by either *F*_ST_ or SPA analyses described above. This may partly result from very different MAFs for *F*_ST_ and SPA outliers relative to MAFs for envGWAS outliers.

In contrast, *F*_ST_ and SPA results produced some overlapping variants. The highest absolute values for SPA involved SNPs 2_02051 and 2_02052 (Table 2), located within the same single gene (*Vigun11g148700*) on Vu11 with these same SNPs also found as *F*_ST_ outliers. Additional SNPs overlapping between *F*_ST_ and SPA were found on Vu04 (2_40290 and 2_40224) and Vu07 (2_03060 and 2_25770). Two SPA outliers on Vu05 (2_05074 and 2_16071) occurred in a region with elevated *F*_ST_ values, but the same variants were not involved.

If *F*_ST_ outliers were driven by temperature and SPA outliers by precipitation, this raises a question of overlap between heat and drought signals. BIO1 and BIO12 showed only weak correlation across the accession panel (Pearson *r* = 0.17, *p* < 0.0001; 95% CI: 0.09–0.25) indicating that temperature and precipitation gradients are largely independent in our sampling.

### 3.6 LD Decay, Variant Annotation and Enrichment Analysis of Hits in Three Functional Categories

Pairwise LD, measured as *r* ^2^, decays rapidly with increasing physical distance, with *r* ^2^ reaching half of its initial value by ~32 kb, while *r* ^2^ = 0.20 was reached at ~150 kb (Figure S9), consistent with a previous LD assessment in a core collection of 2021 cowpea accessions (Fiscus et al. 2024). This relatively rapid decay reflects a compact LD structure across the cowpea genome and implies that association signals are likely to capture variants in close physical proximity to causal loci.

BLAST searches using the contextual sequence around each outlier SNP identified matches in the NCBI GenBank nucleotide database (Sayers et al. 2024) for 150 of 297 variants identified in our various comparative analyses. This analysis was implemented in SNPMeta (Kono et al. 2014). A table of SNPMeta output for each SNP detected as an outlier in our analyses is reported in Table S6. Among the variants producing BLAST results were two SNPs found within heat shock proteins (1_0346 and 2_05343) and three SNPs found within various cytochrome P450 gene family members (2_21837, 2_33252, 2_46982). We also found a histone acetyltransferase GCN5 protein (2_21186), which is a chromatin regulator associated with transcriptional activation under heat stress. Two SNPs mapped to ion transport‐related genes, including Na^+^/H^+^ (2_03060) and K^+^ (2_35005) transporters involved in osmotic regulation and drought stress adaptation, while the SNP 2_32281 was found in the region of a gene encoding the protein phosphatase (PP 2C 32), an ABA‐dependent signalling involved in plant drought response.

We performed an enrichment analysis calculating odds ratio to determine whether there were proportionally more variants in functional categories (genic), coding sequence (CDS), and nonsynonymous than expected relative to the proportions in the full genotyping data. Our 297 outliers from envGWAS, *F*_ST_ and SPA were enriched for SNPs in genic regions (1.13), for CDS (1.24), and for nonsynonymous SNPs (1.33). However, the observed trend was non‐significant in a Fisher's exact test, with the greatest enrichment for CDS resulting in *p* = 0.06 (Table 3).

**TABLE 3:** Enrichment of candidate SNPs within functional categories.

Category	Candidate SNPs (in category)	Candidate SNPs (out category)	All SNPs (in category)	All SNPs (out of category)	Odds Ratio	*p*
Genic	195	102	27,710	16,396	1.13	0.17
CDS	79	218	9977	34,129	1.24	0.06
Nonsynonymous	37	260	4272	39,834	1.33	0.07

*Note:* SNPs in candidate = Number of SNPs in the category in the candidate list. SNPs out candidate = Number of SNPs not in the category in the candidate list. SNPs in full data = Number of SNPs in the category in the full data. SNPs out full data = Number of SNPs not in the category in the whole dataset. Odds Ratio = (SNPs in candidate × SNPs out full data)/(SNPs out candidate × SNPs in full data).

### 3.7 Ancestral State Inference

Alignments of outgroup samples to the cowpea reference genome were most complete when using the minimap2 setting ‘ASM20’. The nucleotide states for *V. unguiculata* iSelect SNPs were identified by querying the assemblies of three cowpea relatives aligned to the cowpea reference genome. This resulted in the identification of nucleotide state for 12,869 SNPs for *V. vexillata* ; 10,955 for *V. trilobata* and 10,281 for *V. marina* . Using estSFS (Keightley and Jackson 2018) for maximum‐likelihood inference of ancestral state, 29,218 SNPs were identified with an inferred probability of correct inference of ancestral states of > 80%. The alternate allele was determined to be ancestral for 15,322 SNPs, with the reference allele ancestral for 13,896 SNPs. The ancestral state of each selected SNP is indicated in Table 1 (*F*_ST_ outliers), Table 2 (SPA outliers) and Table S5 (envGWAS outliers).

## 4 Discussion

Based on envGWAS, we identified 98 variants that survived FDR correction for association with heat‐related variables. There were significant associations on all chromosomes, with the largest number of variants identified on chromosomes Vu03 (13 SNPs) and Vu04 (12 SNPs). Both *F*_ST_ and SPA analysis identified variants that are also putatively associated with adaptive differences across the species range. The comparison of alternate allelic states to environmental variables shows that all the *F*_ST_ were most strongly associated with temperature differentiation, while SPA outliers were associated with precipitation differences. This difference reflects the way outliers are defined in these two approaches. By delineating samples on either side of 12° N latitude, we divided cowpea lines from hot semi‐arid climates and hot desert regions in the southern Sahara from those from the tropical savannah, essentially forcing outlier allele frequency to occur as differences in temperature. This has the desirable effect of identifying variants that occur more frequently in lines from hotter environments. However, because SPA identifies gradients in allele frequency without arbitrary partitions of the sample, it could be argued that the differentiation in allele frequency identified may be more closely associated with selection imposed by the environment in shaping allele frequencies. That is, selection for differences in precipitation may have been the more important adaptive force in cowpea. This observation appears consistent with a comprehensive environmental association study across 25 plant species, which concludes that precipitation‐related variation is more frequently observed than temperature‐related variation (Whiting et al. 2024). This may be partly because temperature changes have occurred more recently, resulting in fewer generations of selective pressure on adaptation to increasing temperature within environments (Whiting et al. 2024).

We identified a much larger number of variants significantly associated with precipitation‐related environmental variables than with heat‐related variables. This resulted in 72 of the 149 precipitation‐related variants also detected as outliers for heat‐related variables (Figure 5). *Prima facie*, this suggested that precipitation differences across the geographic range of our sample were important determinants of allelic differentiation among samples, and perhaps also that these traits were inextricably linked. While it is difficult to infer the physiological basis of heat stress from these results, many variants with the strongest association with heat stress are also associated with water availability (Figure 5).

As noted above, envGWAS and the allele frequency outlier approaches identified largely independent sets of variants. However, *F*_ST_ and SPA results overlapped, particularly for some extreme allele frequency outliers. At times, a lack of overlap among variants detected across these approaches has led to speculation about the veracity of the results. For example, Anderson et al. (2016) found little overlap among the same comparisons, that is, envGWAS, *F*_ST_ and SPA, in a study of *Glycine soja* , the closest wild relative of cultivated soybean ( *Glycine max* ). However, Lei et al. (2019) in seeking to determine if previously cloned genes involved in environmental differentiation in barley landraces, showed potential evidence of selective history in envGWAS and allele frequency comparisons, found many well‐characterised genes identified as significant based on envGWAS, one of several *F*_ST_ comparisons, or both. Several well‐characterised genes with known effects in barley were identified across multiple comparisons. For example, Phytochrome C, a flowering time‐related locus, was identified using four bioclimatic variables and an elevation *F*_ST_ comparison (Lei et al. 2019). Other well‐characterised genes were identified using only one of the approaches, suggesting that the approaches are at times complementary (Campbell et al. 2025). These results may also reflect some of the challenges in detecting variants, including factors such as SNP density (Lei et al. 2019) and the complexity of environmental factors that can drive adaptation (Lotterhos 2023).

Approaches for improving the detection of variants associated with adaptation to the environment are being actively explored (Campbell et al. 2025). One approach exploits linkage disequilibrium among linked variants (Booker et al. 2024) to increase the detection of adaptive variants. Our LD analyses show that *r* ^2^ decays relatively rapidly in cowpea, reaching half of the initial value within 32 kb. Given the relatively small and gene‐dense genome of cowpea, genotyped markers could capture signals from nearby causal variants through linkage disequilibrium, facilitating the identification of associations within relatively small genomic intervals (Bush and Moore 2012). This LD scale served as a proxy for our WZA analysis, in which we used a 50 kb window to aggregate signals across physically linked markers. We identified 617 outlier windows. Interestingly, 41 of our SNP outliers overlapped 24 of these windows, indicating that SNP‐level signals are not isolated statistical artefacts but could reflect broader genomic regions responding to environmental pressures (Hoban et al. 2016).

The four analytical approaches used here are largely complementary, potentially capturing different facets of how environmental gradients could shape nucleotide frequency differences across the sampled geographic/climatic range. Our primary *F*_ST_ comparison involved clearly contrasting climatic categories, thus maximising potential to identify marked allele‐frequency differentiation that has value for conservation and breeding (Figure 3). The temperature differences between climate categories are quite distinct and *F*_ST_ outliers identified large differences in geographies, including variants that are clearly localised to warmer climates. SPA detected steep spatial gradients in allele frequency, independently of predefined groupings of samples. This analysis found the steepest allele frequency gradients for variants that our ranking‐based approach identifies as most closely related to precipitation variables. EnvGWAS is particularly valuable because it highlighted associations with specific climatic variables while WZA aggregated signals across linked markers to show region‐level signatures that may be missed at the single‐SNP scale. The fact that these approaches are sensitive to different aspects of the genomic structure and environmental variations explains the partial overlap among outliers and suggests the importance of using multiple methods when exploring the evolution of local adaptation. Loci identified by several of them thus represent the strongest candidates for climatic adaptation (Figure 5). Thus, the resulting list of candidate genomic regions is useful for further exploration of underlying variation and produces an actionable set of candidate loci and variants for resequencing, for experimental validation as well as for operationalisation within breeding programs.

There is considerable potential to use our results to identify cowpea genetic resources likely to carry variants relevant to increased heat tolerance in cowpea lines grown as a critical food source in regions where heat stress is likely to become increasingly important. This process could begin with a focus on variants detected here that occurred at a relatively low frequency among current breeding materials from programs in West Africa (Muñoz‐Amatriaín et al. 2017) and extend to machine‐learning‐based methods that enable the annotation of variants beyond coding regions (Benegas et al. 2023; Zhai et al. 2024).

## Author Contributions

R.A., E.J.L., J.B.P., K.M.V., H.A., M.M.‐A. and P.L.M. performed data analysis. R.A., J.B.P., K.M.V. and P.L.M. wrote the manuscript. R.A., M.M.‐A., E.J.L., M.K.B., L.G., E.F.R., O.B., K.J.B. and P.L.M. designed the research. E.F.R., K.J.B., M.M.‐A., O.B. and P.L.M. acquired funding. All authors reviewed and edited the manuscript.

## Funding

This work was supported by the Foundation for Food and Agriculture Research, ICRC20‐0000000032.

## Conflicts of Interest

The authors declare no conflicts of interest.

## Supporting information


**Figure S1:** (a) Pearson pairwise correlation matrix of Bioclim variables. The heatmap represents correlation coefficients between the 19 Bioclim variables. The red indicates strong positive correlations and blue indicates strong negative correlations. The colour gradient ranges from −1.0 (strong negative correlation) to 1.0 (strong positive correlation). (b) The density hexagonal binning plot shows relationship between mean annual temperature (BIO1) and annual precipitation (BIO12). Each hexagon represents the count of populations within that environmental range. Colours transition from blue to red, with deeper reds indicating areas of higher population density.
**Figure S2:** Location verification results for cowpea accessions. Red points indicate geographic coordinates from the original passport file, while the green points show the verified or updated geolocation for the accession. Yellow lines show paths between original and updated locations.
**Figure S3:** A depiction of the Köppen‐Geiger climatic zone across the African continent. The collection locality of cowpea accessions is shown. The line at 12° N latitude indicates the partition used for allele frequency comparisons.
**Figure S4:** A plot of likelihood value for STRUCTURE analysis across a range of population numbers (*K*) from 2 to 7. The best fit to cowpea genotyping data occurs at *K* = 5.
**Figure S5:** Principal Component Analysis (PCA) showing genetic clustering following Principal components 1 and 3. ATS = African Tropical Savannah (Yellow), WAAS = West African Arid Steppe (Red), CWAT = Coastal West African Tropical (Light blue), SEA = Southeastern Africa, (Green), NAD = North African Desert (Dark blue).
**Figure S6:** A workflow depicting outlier detection approaches used for detection of variants putatively associated with environmental adaptation in cowpea. The approaches in allele frequency comparisons (*F*_*ST*_), spatial ancestry analysis (SPA) and genome‐wide association analysis using environmental variables (envGWAS).
**Figure S7:** A comparison of the allele frequencies for the SPA outlier 2_30853 with annual precipitation. The map represents the annual mean precipitation from 1970 to 2000, with white indicating low precipitation and blue indicating high precipitation (ranging from 0 to 11,250 mm). Alleles for variant 2_30853 are plotted on the map: white dots represent the reference allele (G), and black dots represent the alternate allele (T).
**Figure S8:** A comparison of the allele frequencies for the SPA outlier 2_42020 with annual precipitation. The map represents the annual mean precipitation from 1970 to 2000, with white indicating low precipitation and blue indicating high precipitation (ranging from 0 to 11,250 mm). Alleles for variant 2_42020 are plotted on the map: white dots represent the reference allele (C), and black dots represent the alternate allele (T).
**Figure S9:** LD decay in cowpea. Scatterplot and LOESS‐smoothed curve show the decline of pairwise *r* ^2^ with physical distance between SNPs. LD decays rapidly, reaching half of its initial value at approximately 32 kb.

**Table S1:** mec70327‐sup‐0002‐TableS1.csv.

**Table S2:** mec70327‐sup‐0003‐TableS2.csv.

**Table S3:** mec70327‐sup‐0004‐TableS3.csv.

**Table S4:** mec70327‐sup‐0005‐TableS4.csv.

**Table S5:** mec70327‐sup‐0006‐TableS5.csv.

**Table S6:** mec70327‐sup‐0007‐TableS6.csv.

## Data Availability

Genotyping data was derived from the cowpea Illumina iSelect genotype platform and was previously reported (Fiscus et al. 2024) and can be downloaded from https://doi.org/10.6086/D19Q37. Genotyping data used in this study is available in PLINK and VCF format, using updated physical positions of variants as reported by Liang et al. (2024). Files are available from the Data Repository for University of Minnesota (DRUM) at https://doi.org/10.13020/XXPS‐Q694. A Keyhole Markup Language (KML) file is provided for sample localities and can be used to display localities using Google Earth. All code used for data analysis and figure generation is available from https://github.com/MorrellLAB/cowpea_environmental.
