# Supplementary material for: Identification of Genetic Variation Associated With Heat Tolerance in Cowpea ( Vigna unguiculata L. Walp.)
Source: Mol Ecol. 2026 Apr 3;35(7):e70327. doi: 10.1111/mec.70327 (PMC13047730; doi:10.1111/mec.70327)
Supplement: Supplementary file 1 — Figure S1: (a) Pearson pairwise correlation matrix of Bioclim variables. The heatmap represents correlation coefficients between the 19 Bioclim variables. The red indicates strong positive correlations and blue indicates strong negative correlations. The colour gradient ranges from −1.0 (strong negative correlation) to 1.0 (strong positive correlation). (b) The density hexagonal binning plot shows relationship between mean annual temperature (BIO1) and annual precipitation (BIO12). Each hexagon represents the count of populations within that environmental range. Colours transition from blue to red, with deeper reds indicating areas of higher population density. Figure S2: Location verification results for cowpea accessions. Red points indicate geographic coordinates from the original passport file, while the green points show the verified or updated geolocation for the accession. Yellow lines show paths between original and updated locations. Figure S3: A depiction of the Köppen‐Geiger climatic zone across the African continent. The collection locality of cowpea accessions is shown. The line at 12° N latitude indicates the partition used for allele frequency comparisons. Figure S4: A plot of likelihood value for STRUCTURE analysis across a range of population numbers (K) from 2 to 7. The best fit to cowpea genotyping data occurs at K = 5. Figure S5: Principal Component Analysis (PCA) showing genetic clustering following Principal components 1 and 3. ATS = African Tropical Savannah (Yellow), WAAS = West African Arid Steppe (Red), CWAT = Coastal West African Tropical (Light blue), SEA = Southeastern Africa, (Green), NAD = North African Desert (Dark blue). Figure S6: A workflow depicting outlier detection approaches used for detection of variants putatively associated with environmental adaptation in cowpea. The approaches in allele frequency comparisons (F ST ), spatial ancestry analysis (SPA) and genome‐wide association analysis using environmental variables (envGWAS [file MEC-35-e70327-s001.docx]

Identification of genetic variation associated with heat tolerance in cowpea (*Vigna unguiculata* L. Walp.)

Akakpo Roland^1^, Lee Elaine J^1^, Pacheco Jacob B^1^, Rios Esteban F^2^, Kant ar Michael B^3^, Boukar Ousmane^4^, Volz Kevin M^5^, Akinmade Habib^6^, Getino Luis^7^, Boote Kenneth J^8^, Muñoz-Amatriaín María^7^, Morrell Peter L^1^

**Supplemental figures**

**
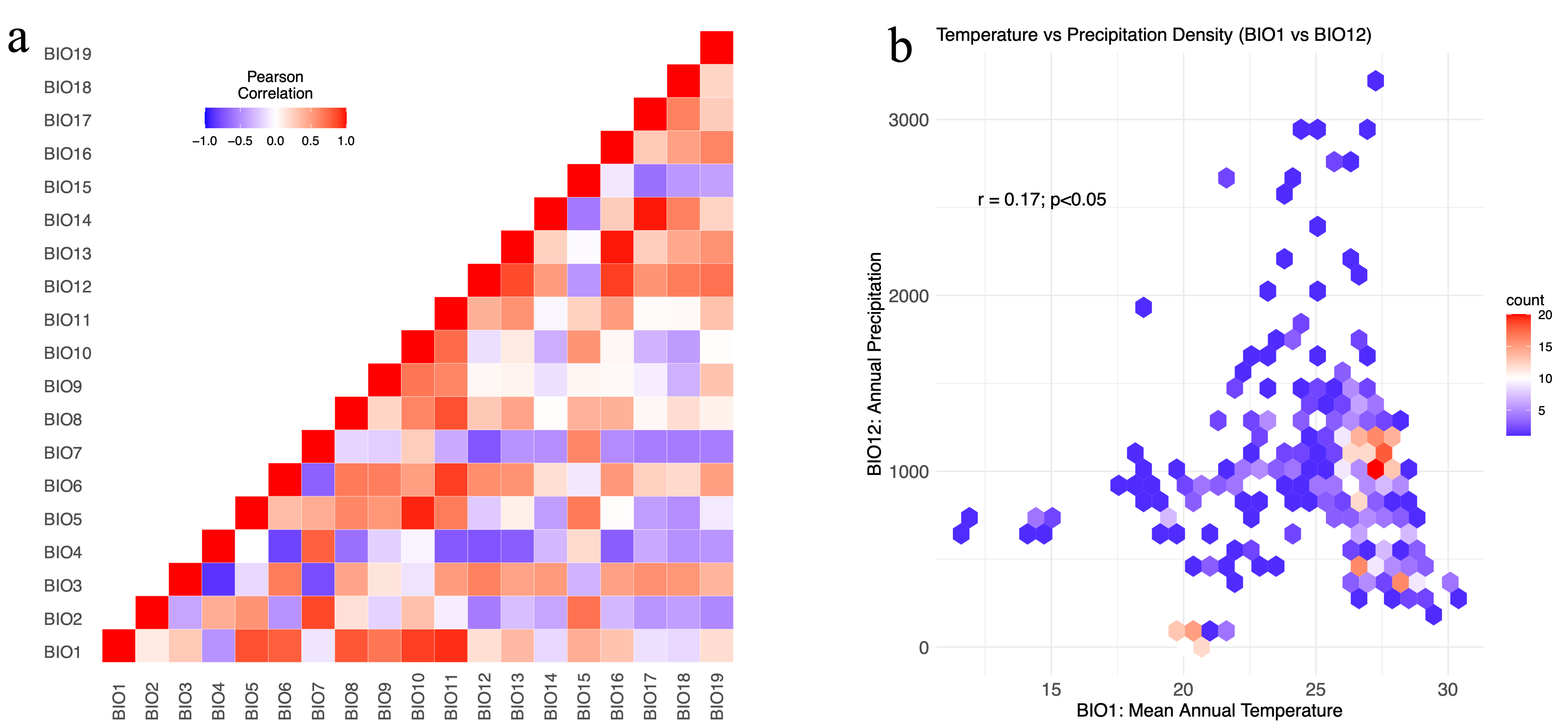
**

**Figure S1.** (a) Pearson pairwise correlation matrix of Bioclim variables. The heatmap represents correlation coefficients between the 19 Bioclim variables. The red indicates strong positive correlations and blue indicates strong negative correlations. The color gradient ranges from -1.0 (strong negative correlation) to 1.0 (strong positive correlation). (b) The density hexagonal binning plot shows relationship between mean annual temperature (BIO1) and annual precipitation (BIO12). Each hexagon represents the count of populations within that environmental range. Colors transition from blue to red, with deeper reds indicating areas of higher population density.


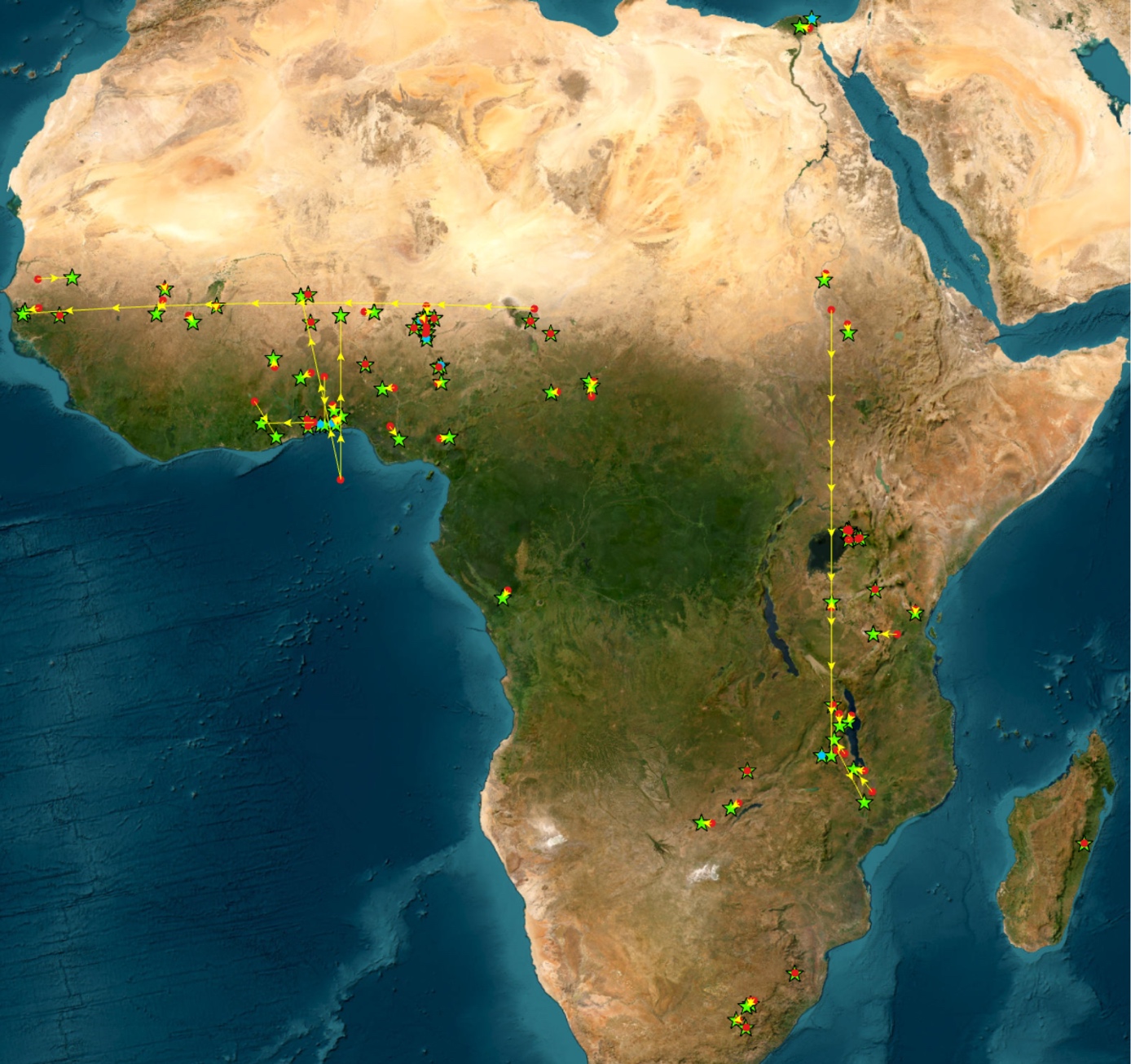


**Figure S2.** Location verification results for cowpea accessions. Red points indicate geographic coordinates from the original passport file, while the green points show the verified or updated geolocation for the accession. Yellow lines show paths between original and updated locations.


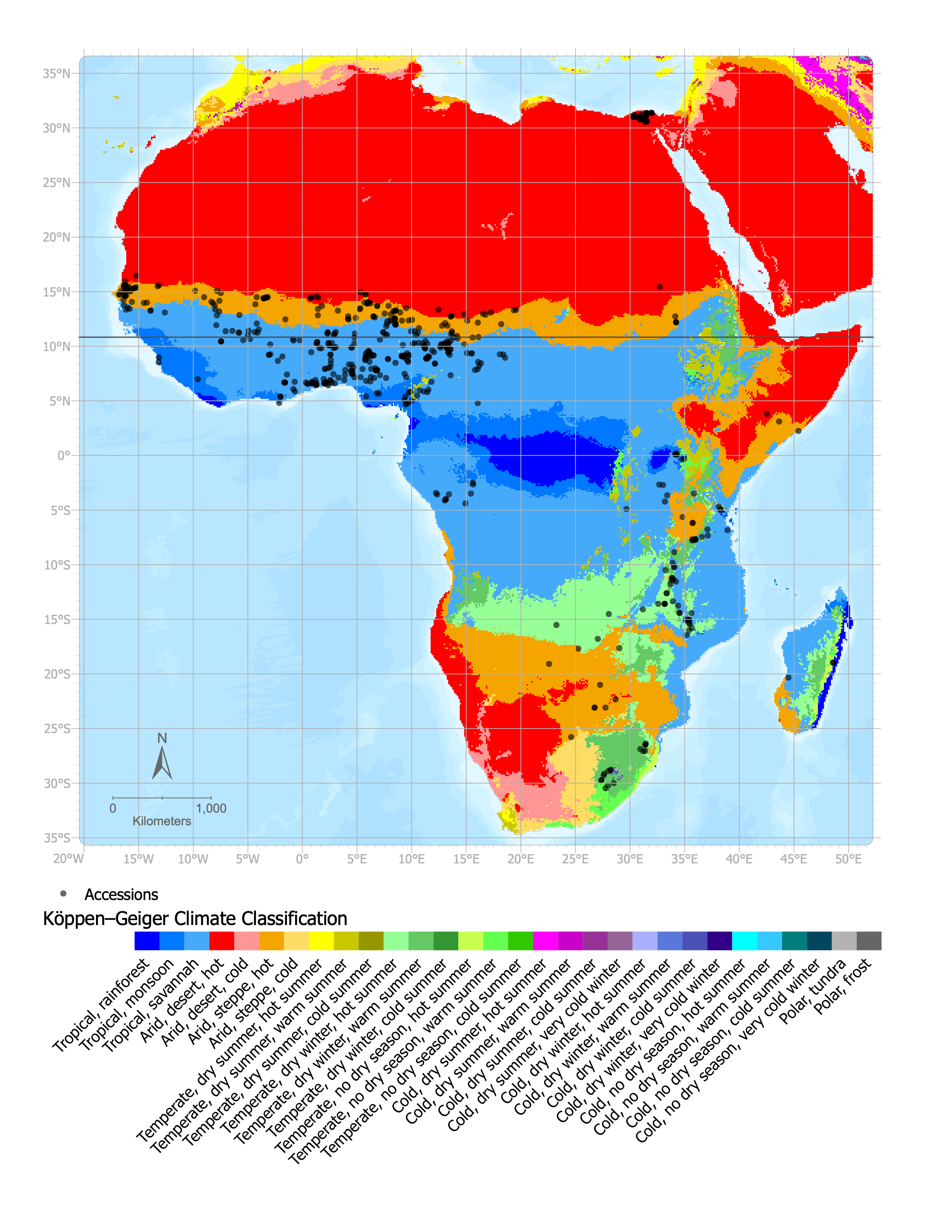


**Figure S3.** A depiction of the Köppen-Geiger climatic zone across the African continent. The collection locality of cowpea accessions is shown. The line at 12˚N latitude indicates the partition used for allele frequency comparisons.

**
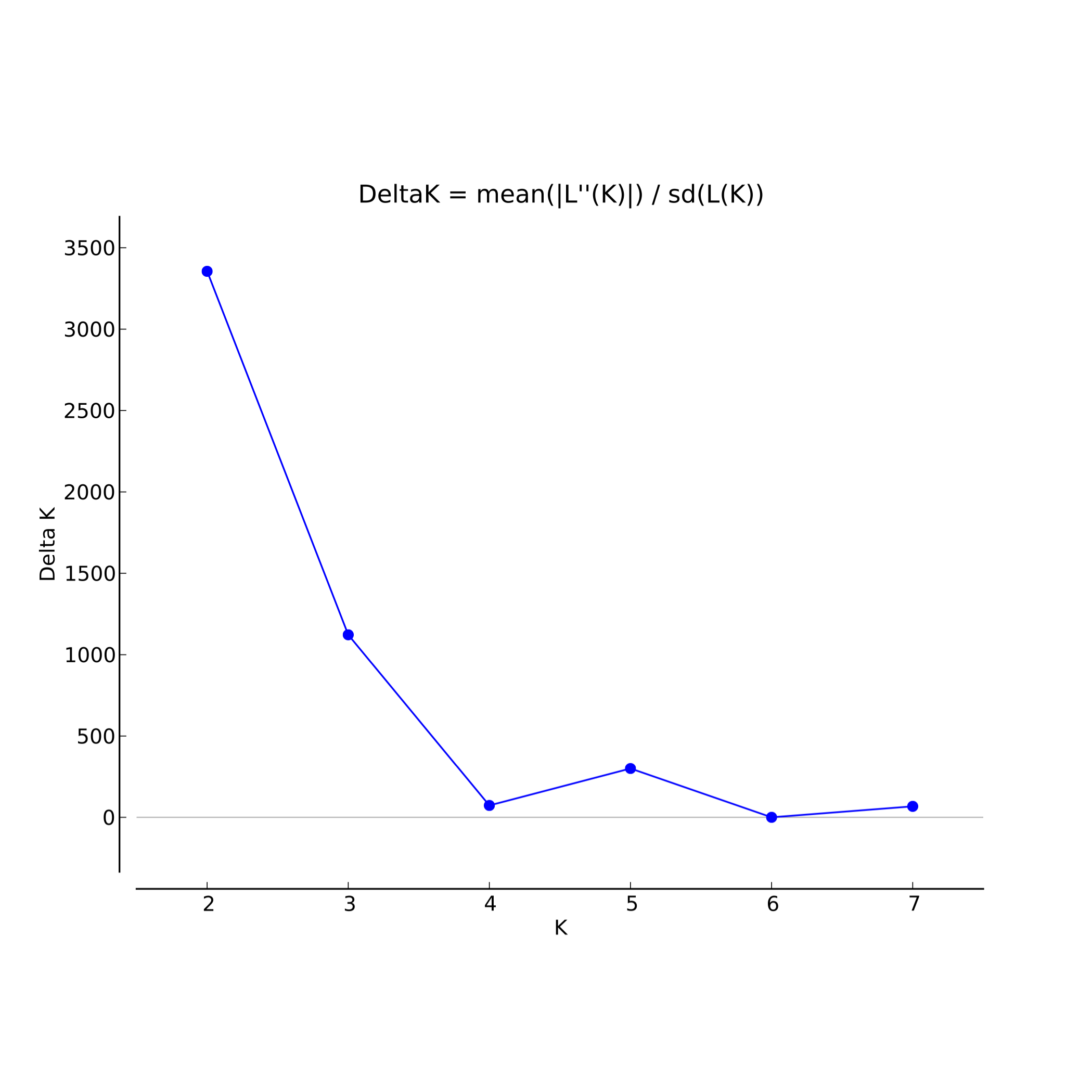
**

**Figure S4.** A plot of likelihood value for STRUCTURE analysis across a range of population numbers (*K*) from 2 to 7. The best fit to cowpea genotyping data occurs at *K*=5.


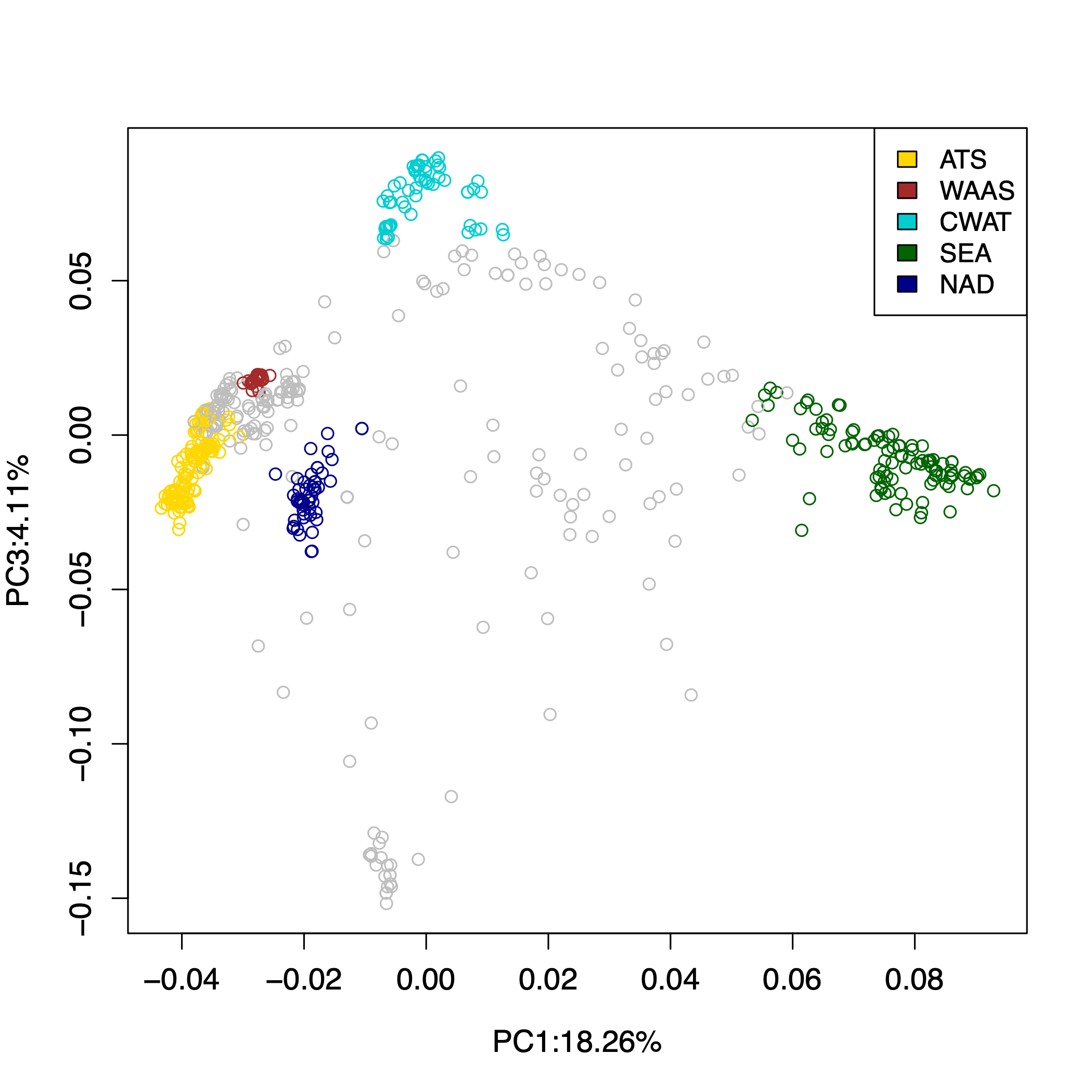


**Figure S5.** Principal Component Analysis (PCA) showing genetic clustering following Principal components 1 and 3. ATS = African Tropical Savannah (Yellow), WAAS = West African Arid Steppe (Red), CWAT = Coastal West African Tropical (Light blue), SEA = Southeastern Africa, (Green), NAD = North African Desert (Dark blue).

**
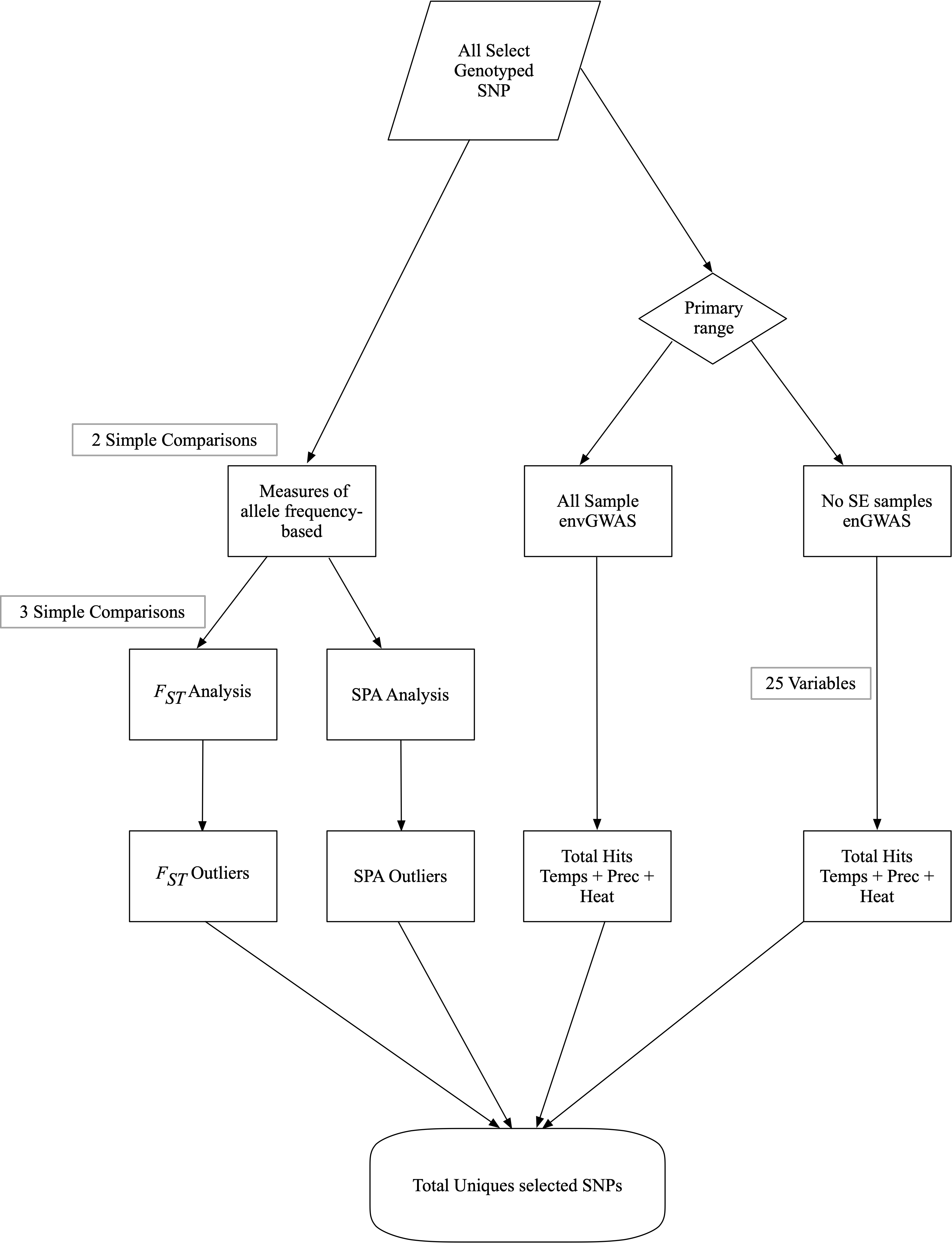
**

**Figure S6.** A workflow depicting outlier detection approaches used for detection of variants putatively associated with environmental adaptation in cowpea. The approaches in allele frequency comparisons (*F_ST_*), spatial ancestry analysis (SPA), and genome-wide association analysis using environmental variables (envGWAS).

**
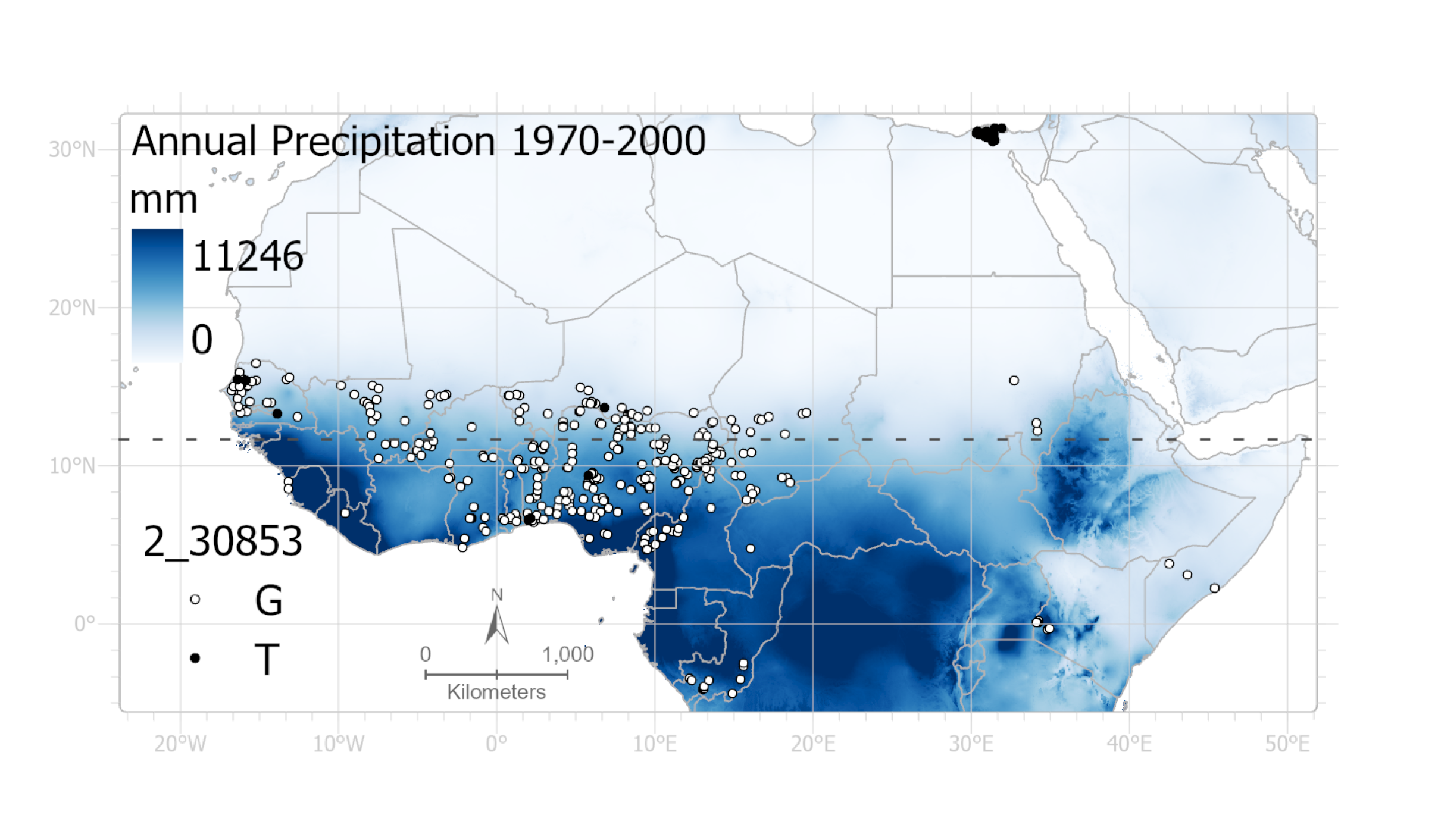
**

**Figure S7.** A comparison of the allele frequencies for the SPA outlier 2_30853 with annual precipitation. The map represents the annual mean precipitation from 1970 to 2000, with white indicating low precipitation and blue indicating high precipitation (ranging from 0 to 11250mm). Alleles for variant 2_30853 are plotted on the map: white dots represent the reference allele (G), and black dots represent the alternate allele (T).


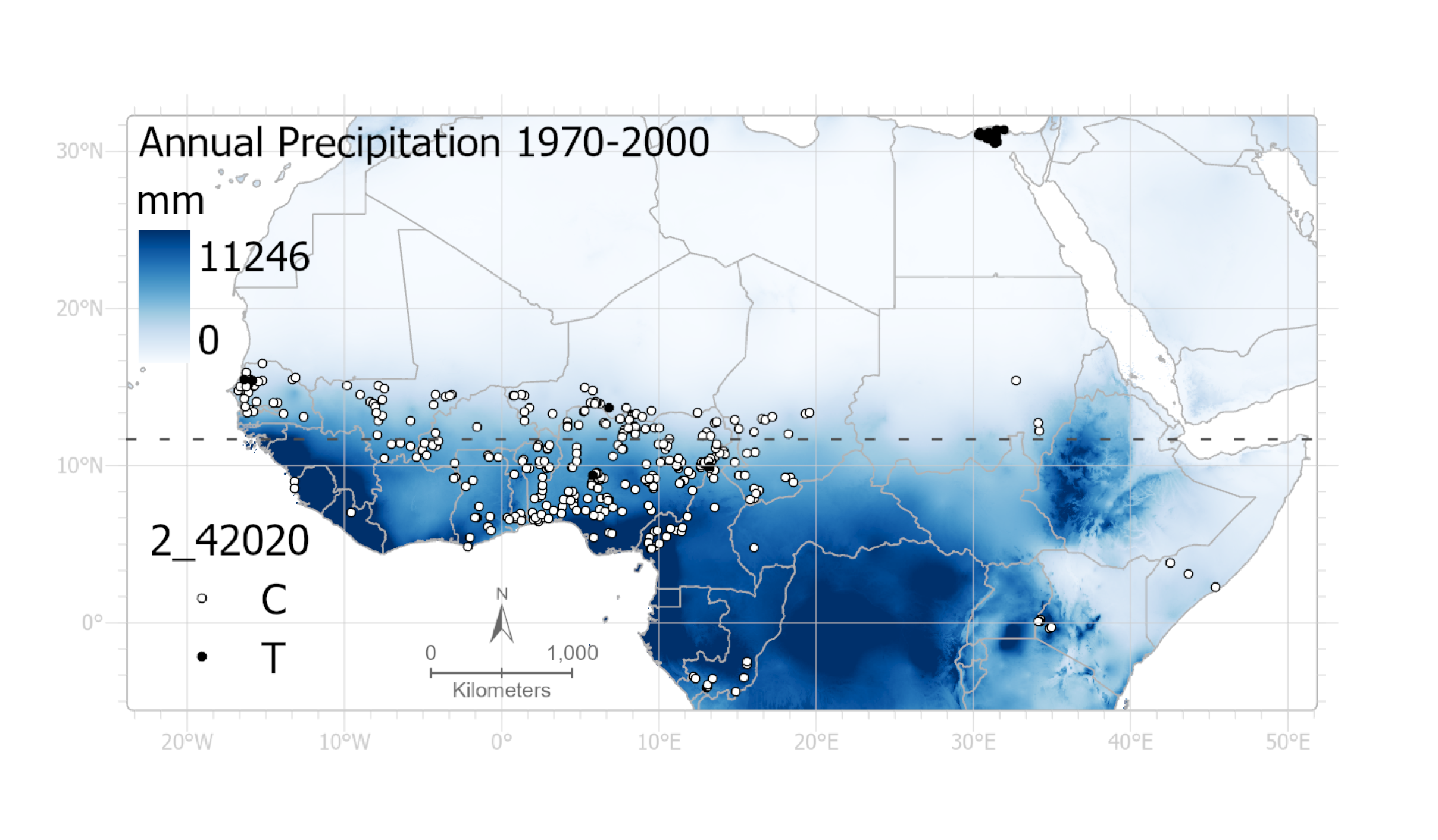


**Figure S8.** A comparison of the allele frequencies for the SPA outlier 2_42020 with annual precipitation. The map represents the annual mean precipitation from 1970 to 2000, with white indicating low precipitation and blue indicating high precipitation (ranging from 0 to 11250mm). Alleles for variant 2_42020 are plotted on the map: white dots represent the reference allele (C), and black dots represent the alternate allele (T).


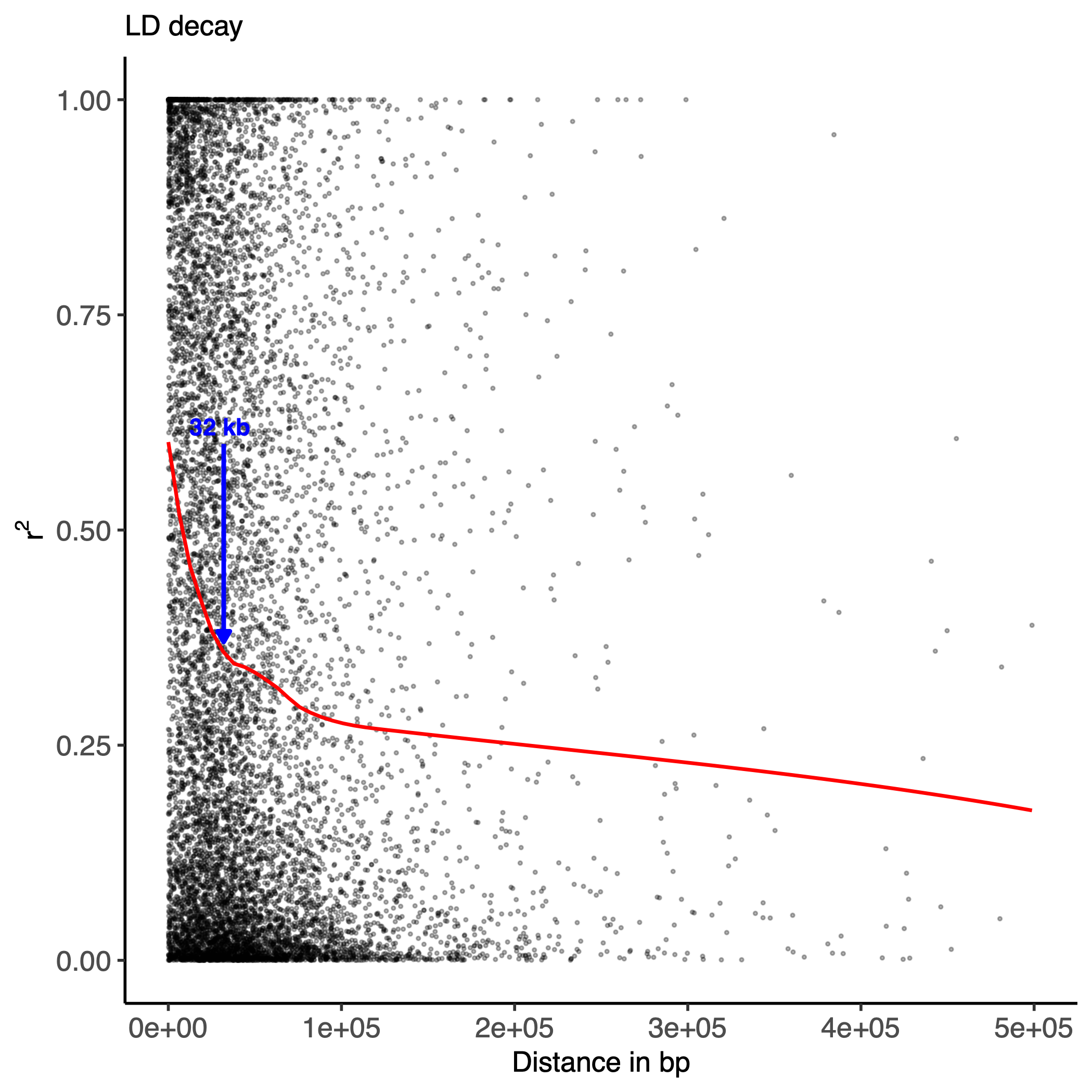


**Figure S9.** LD decay’s state in cowpea. Scatterplot and LOESS-smoothed curve show the decline of pairwise *r*² with physical distance between SNPs. LD decays rapidly, reaching half of its initial value at approximately 32 kb.
